# Micro/nanoplastics pollution: emerging challenges for aquatic animals and food crops

**DOI:** 10.3389/ftox.2026.1768236

**Published:** 2026-03-26

**Authors:** Shrutarshee Kundu, Neeraj Kumar, Paritosh Kumar, Basavaraj PS, Ajay Kumar Singh, Dipti Godse, Kotha Sammi Reddy

**Affiliations:** ICAR-National Institute of Abiotic Stress Management, Baramati, Pune, India

**Keywords:** emergent contaminants, fish, micro/nanoplastics, rice, stress

## Abstract

Recent reports from worldwide reveal that micro/nanoplastics (MNPs) are pervasive pollutants affecting all ecosystems and a wide range of organisms, including animals, plants, fish, humans, and microorganisms. MNPs have been detected in food items, mother milk, vegetables, and other consumable products, indicating their potential to impact organisms across all life stages. These particles can enter the body through inhalation, ingestion, and dermal contact. Due to their small size, micro/nanoplastics can be readily absorbed by animals and plants, leading to adverse effects on human health and ecological integrity. The present review addresses recent concerns related to MNPs pollution in aquatic animals and crops, with a particular focus on fish and rice. Exposure to MNPs has been reported to impair fish growth performance, immune responses, antioxidant status, digestive functions, reproduction, transgenerational effects, endocrine regulation, vitellogenin induction, neurotransmitter activity, and blood biochemical profiles. Similarly, MNPs adversely affect rice production by influencing various stages of the cropping system, including seed germination, vegetative growth, root and shoot development, tillering, and grain yield. Notably, both fish and rice are staple food sources for humans, highlighting the significance of this issue for food safety and public health. This review emphasizes the urgent need for comprehensive studies on the impacts of micro/nanoplastics on aquatic animals and major food crops. It integrates systematic knowledge on the effects of MNPs on fish growth patterns, immunity, endocrine disruption, reproduction, and key physiological indices, as well as on rice growth and productivity. The synthesized information will be highly valuable for policymakers, government agencies, pollution control authorities, and other stakeholders in policy formulation and decision-making processes.

## 1 Introduction

Pollution poses a major threat to aquatic ecosystems, with emerging contaminants such as micro/nanoplastics (MNPs), heavy metals, and pesticides adversely affecting the entire life cycle of aquatic organisms, including fish (Mustafa et al., 2024). MNPs are particularly harmful, not only to aquatic organisms but also across ecosystems, impacting crops, animals, fish, and humans (Habumugisha et al., 2024). Although plastics have become an integral part of modern life, their improper disposal, fragmentation, and degradation have contributed to the increasing prevalence of MNPs in the environment. These particles include both primary MNPs, which are intentionally manufactured at the micro or nanoscale, and secondary MNPs, which are generated through the breakdown of larger plastic items (Habumugisha et al., 2022; Kumar et al., 2023). The emergence of these minute plastic particles has raised significant concerns due to their persistence in water bodies and their potential adverse effects on aquatic organisms (Rehman et al., 2024) as well as crops (Yang et al., 2023). Microplastics are generally defined as plastic particles smaller than 5 mm, originating from both primary and secondary sources (Horton et al., 2017). They consist of a heterogeneous mixture of materials with varied structures, including fragments, fibres, filaments, beads, spheres, sheets, films, and pellets, ranging in size from 0.1 µm to 5,000 µm (Lusher et al., 2017a). Over time, these microplastics further degrade into nanoplastics. Due to their small size and unique physicochemical properties, MNPs pose a greater environmental risk than larger plastic debris (Zhang et al., 2020) and are now recognized as an emerging environmental pollutant of global concern. The distribution of MNPs within the food web has important ecological implications. Phytoplankton, which forms the base of aquatic food webs, shows the highest uptake and bioaccumulation of MNPs (Benson et al., 2022; Thomas et al., 2021). These contaminants are subsequently transferred to higher trophic levels through consumption by zooplankton, small aquatic organisms, fish, and ultimately humans (Khanashyam et al., 2023). Human exposure to MNPs through food and water has raised serious health concerns (Galloway, 2015). Moreover, the accumulation of MNPs may disrupt aquatic ecosystem dynamics, leading to altered population structures, shifts in species composition, and potential biodiversity loss (Gomes et al., 2022).

Micro/nano nanoplastics (MNPs) have been shown to adversely affect aquatic organisms, including fish, by reducing growth, impairing reproduction, and disrupting overall physiological processes (Habumugisha et al., 2026). Fish can be exposed to MNPs through several pathways, including direct ingestion, in which plastics are mistakenly perceived as food (Worm et al., 2017); accidental consumption; or indirect intake (secondary ingestion) through prey that has already ingested these particles (Watts et al., 2014). Moreover, microplastics (MPs) often contain additives introduced during the manufacturing process and have a high capacity to absorb harmful pollutants, including persistent, bio-accumulative, and toxic substances, from the surrounding environment (Martín et al., 2023; Yu et al., 2022; Stapleton and Hai, 2023; Weichselbaum and Klein, 2018). It is well established that MNPs can persist in the gastrointestinal tract (GIT) of aquatic fauna for several days or even up to a week, during which they may be ingested, retained, or eventually eliminated through excretion (Ghosh, 2025; Yang et al., 2023). The retention of MNPs in the GIT provides opportunities for their translocation to other body tissues or their transfer along the food chain Exposure to MNPs, even within a single fish species, can adversely affect feeding efficiency, survival, reproductive performance, and growth due to factors such as clogged feeding structures or reduced prey consumption (Foley et al., 2018; Yang et al., 2023). In severe cases, physical damage such as stomach deformation or rupture may occur, which can be fatal. Furthermore, exposure to MNPs has been associated with multiple deleterious effects in fish, including reduced growth and reproductive success, endocrine disruption, cellular and tissue lesions, oxidative stress, metabolic dysfunction, immunosuppression, and genotoxicity (Ghosh, 2025).

Beyond aquatic systems, MNPs contamination has emerged as a significant concern for agroecosystems, particularly rice, a globally important staple grown on approximately 170 million hectares and consumed by more than half of the world’s population (Yang and Gao, 2022). Increasing pollution has subjected rice cultivation to multiple abiotic stressors, including heavy metals, plastic debris, nutrient deficiencies, drought, and salinity, which collectively threaten productivity across developmental stages (Liu et al., 2022a). Recent studies have reported substantial MNPs contamination in paddy soils, with concentrations ranging from 10^3^ to 10^4^ particles kg^-1^ (Katsumi et al., 2021). Rice plants can readily take up micro- and nanoplastics from soil and translocate them to above-ground tissues, including grains, raising concerns for food safety (Liu et al., 2022a). Comparative studies suggest that rice is more susceptible to MNPs uptake than wheat (Roy et al., 2023), with uptake efficiency largely governed by the particles’ physicochemical properties, including size, shape, surface charge, and polymer type (Spano et al., 2022).

**FIGURE 1:**
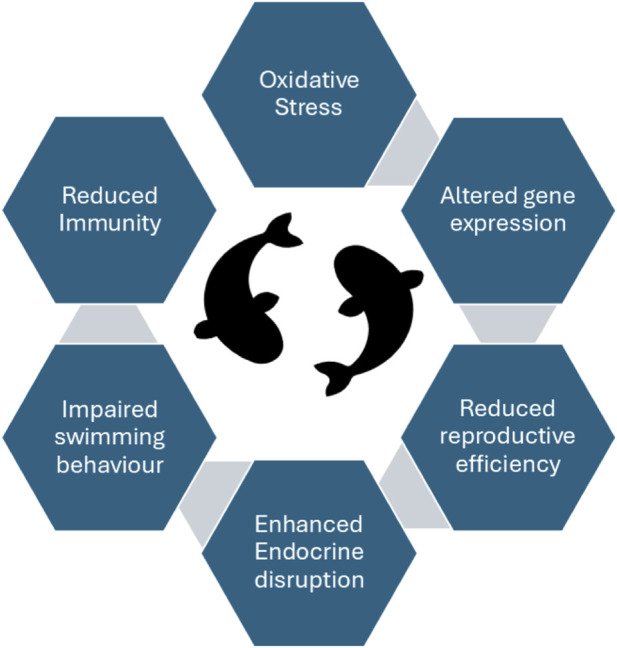
Effect of Micro/nanoplastics (MNPs) on fish.

The present review focuses on MNPs pollution in aquatic animals, particularly fish, and in a globally important crop, rice. It synthesizes current evidence on the effects of MNPs exposure in fish, including impacts on growth performance, immune responses, oxidative stress, morphological alterations, reproductive success, gene regulation, genotoxicity, metabolic function, and neurotoxicity. In rice, the review examines the influence of MNPs on different growth stages, nutrient uptake, physiological and biochemical attributes, oxidative stress responses, gene expression, nitrogen metabolism, and structural integrity. Figure 1 summarizes the major effects of MNPs on fish. Finally, the review outlines key knowledge gaps and future research directions aimed at improving the management and mitigation of MNPs pollution in both aquatic and agricultural systems.

## 2 Methodology

The present review paper addresses micro/nanoplastics (MNPs) pollution in aquatic animals and rice crops. A comprehensive literature search was conducted using electronic databases such as Google, Scopus, Web of Science, ScienceDirect, PubMed, and Google Scholar. Relevant peer-reviewed research articles, review papers, and reports were identified using specific keywords and Boolean operators. In total, more than 220 references were consulted for this review. During the literature search, keywords such as “micro/nanoplastics (MNPs) pollution in fish*”* were used. The search encompassed various thematic areas, including the types of MNPs and their effects on growth performance, oxidative stress, reproduction, endocrine systems, immunity, and hematological parameters in fish. In addition, literature was also reviewed on the effects of MNPs on rice, covering different growth stages such as seed germination, early growth and development, shoot and root growth, tillering stage, and grain yield. Furthermore, studies addressing the interactions of MNPs with other contaminants were also included.

## 3 Types of micro/nanoplastics

Microplastics are generally classified into two categories based on their origin: primary and secondary. Primary microplastics are small plastic particles intentionally manufactured for use in industrial products such as cosmetics and pharmaceuticals. Common examples include polyethylene (PE), polypropylene (PP), and polystyrene (PS) particles, which are frequently found in cosmetics and medical items (Horton et al., 2017). In contrast, secondary microplastics are formed when larger plastic waste is broken down by physical, chemical, and biological processes (Thompson et al., 2009; Ryan, 2019). During this degradation, plastics may lose their structural integrity through fragmentation and disintegration, eventually becoming invisible to the naked eye. In addition to their origin, microplastics are also categorized based on their physical characteristics, such as shape, size, and chemical composition. Their shapes can vary from spherical to highly irregular forms. Moreover, microplastics often contain a range of additives, including stabilizers, plasticizers, and flame retardants, which can leach into the environment and pose significant risks to both ecosystems and human health (Hu et al., 2023).

## 4 Effects of micro/nanoplastics on aquatic environment

Microplastics is a widespread pollutant in the marine environment, commonly dispersed throughout the oceans, and their ingestion by marine species has raised serious concerns about potential harmful effects. A wide variety of plastic polymers, including polyethylene (PE), polypropylene (PP), low-density polyethylene (LDPE), high-density polyethylene (HDPE), polystyrene (PS), polyvinyl chloride (PVC), polyethylene terephthalate (PET), polyoxymethylene (POM), polybutylene adipate terephthalate (PBAT), and polylactic acid (PLA), have been detected in both marine and terrestrial ecosystems (Khalid et al., 2020). The primary and secondary microplastics originating from multiple sources are present in the aquatic environment (Essel et al., 2015). Several studies have identified aquaculture-related sources of microplastics, including contaminated salt, fishing gear, degraded fish-farming tanks and infrastructure, fish feed, and culture water (Hanachi et al., 2019; Pironti et al., 2021). Recent research indicates that microplastics (<20 μm) and nanoplastics (<100 nm) have significant effects on the behavior, metabolism, reproductive success, and physiology of a wide range of marine organisms (Urme et al., 2019). The small size of microplastics increases the likelihood of their ingestion by marine organisms, where they are often mistaken for food. Once ingested, sharp-edged particles can inflict physical damage to the digestive system, leading to inflammation and severe pathological consequences.

Moreover, microplastics act as carriers of toxic substances, including heavy metals and organic pollutants, which can enter the bloodstream and disrupt reproduction, nutrition, and the function of vital organs in aquatic organisms (Golwala et al., 2021). For instance, microplastics were detected in the digestive tracts of Japanese anchovies, consisting mainly of polyethylene and polypropylene fragments, along with beads and filaments derived from fishing gear (Tanaka and Takada, 2016). “Exposure to microplastics significantly inhibits fish growth, survival, and reproductive capacity, while increasing oxidative stress and inducing cellular damage (Wang et al., 2024).

### 4.1 Effect of micro/nanoplastics on growth performance of the fish

The micro/nanoplastics (MNPs) significantly affect fish growth performance in terms of weight gain percentage, feed efficiency, protein efficiency, and specific growth rate (SGR). The details of these effects are summarized in Table 1. Micro/nanoplastics (MNPs) are known to accumulate in the gastrointestinal tract (GIT) of fish, where they obstruct digestive enzyme pathways, impair digestion, and reduce appetite (Lusher et al., 2013; Lusher et al., 2017b). This accumulation disrupts enzymatic functions essential for nutrient utilization, leading to growth retardation. Correspondingly, reduced survival and growth rates have been documented in fish exposed to MNPs (Lai et al., 2021). The gills represent another sensitive target of MNPs induced toxicity, with damage affecting respiratory efficiency and osmotic and ionic balance (Blonc et al., 2023). These combined effects ultimately compromise feeding efficiency, growth, survival, and reproductive performance in aquatic organisms.

**TABLE 1:** Effect of micro/nanoplastics on growth performance of the fish.

S. no	*Species*	Type of micro/Nanoplastics	Size/Dose	Response	References
1	*Labeo rohita*	PS-MPs), dietary inclusion	0, 0.5, 1, 1.5, 2, 2.5% in diet for 90 days feeding	As PS-MP % increased, growth (FW, WG) decreased; SGR decreased; FCR increased. At highest level (2.5%) lowest growth and worst FCR	Rashid et al. (2025a)
2	*Cirrhinus mrigala*	PS-MPs, sunflower meal-based diets	0, 0.5, 1, 1.5, 2, 2.5% MPs; 90 days	Poorer growth, poorer nutrient digestibility, worse body composition	Rashid et al. (2024a)
3	*Catla catla*	PS-MPs), sunflower meal-based diets	0%, 0.5%, 1%, 1.5%, 2% and 2.5% MPs for 90 days	Inhibited growth and decreased nutritional digestibility; lowest weight gain and the highest feed conversion ratio; lower the protein content and increase fat content of body	Rashid et al. (2024b)
4	*Labeo rohita*	Polyethylene glycol microplastics	0 (control), 1, 10, 100 mg/L in water; 45 days exposure	Growth parameters highest in control; significantly reduced at highest exposure (100 mg/L)	Akhtar et al. (2024)
5	*Poecilia reticulata*	MP/NP exposures (MPs at different concentrations	MPs-160 (higher conc.), also NPs; 14 days exposure	No significant difference in survival; only high concentration (MPs-160) showed significantly lower SGR vs. control	Huang et al. (2020)
6	*Oreochromis niloticus*	Low-density polyethylene microplastics (LDPE-MPs), dietary	Diets containing 0, 2, 4, 6, 8, and 10% LDPE-MPs, 60 days feeding	10% LDPE-MPs, WG ∼85.04%, SGR ∼0.68% (likely per day), FCR increased to ∼3.92. Reduced growth, digestibility, body composition at higher exposure	Mahmood et al. (2024)
7	*Catla catla*	Polystyrene microplastics (PS-MPs), dietary in casein/mollusc meal based diets (CM-based diets)	Polystyrene microplastics (PS-MPs), dietary in casein/mollusc meal based diets (CM-based diets)	Increasing PS-MP inclusion resulted in significant decrease in FW (from ∼30.23 g in control to ∼19.17 g at 2.5%), weight gain and SGR reduced; FCR increased (worst FCR ∼2.60) at highest MP diet	Rashid et al. (2025b)
8	*Danio rerio*	Polystyrene nanoplastics, 100 nm particles, waterborne	Exposed to 0, 100, 200, 400 mg/L; from ∼24 hpf (hours post fertilization), duration up to 96 hpf, etc.	At 200–400 mg/L: significantly reduced body length (after 48–72 h), reduced heart rate, delayed hatching, lower survival. Even 100 mg/L showed milder/no effect on some measures but higher concentrations clearly harmful	Rehman et al. (2025)
9	*Oncorhynchus mykiss*	Microplastics (PS, PET, PE microspheres)	Long-term exposure (69 days) from embryo stage or shorter from larvae (29 days) to various types of MPs: pre-production pellets (∼3,000 μm PS and PET), PE microspheres (150–180 µm) etc	Hatching and survival largely unchanged relative to control; some alteration in length gain after long exposure and changes in yolk sac exhaustion; but final size of larvae did not differ from controls under many treatments. So less strong growth suppression in these early life trout under those MP exposures	Jakubowska et al. (2020)
10	*Neogobius melanostomus*	Microplastic exposure + elevated temperature (combined stressors)	Environmentally relevant concentrations of microplastics; also warming treatments; duration not extremely long but sufficient to measure SGR changes	Microplastics by itself significantly reduced SGR; warming also reduced SGR; combined stressors produced greater reductions in growth. For example, under warming + MP exposure, reductions in SGR (percent daily mass change) of ∼0.29–0.33 vs. ∼0.18 under warming with no MP exposure	D'Avignon et al. (2023)
11	*Paralichthys olivaceus*	Exposure: Polystyrene microplastics	Exposure: Polystyrene microplastics (size ∼197.3 ± 11.2 µm) in marine exposure for 28 days	Growth result: Body length growth was *not* significantly different, but weight growth was substantially slower: in control, weight increased ∼36.3% over 28 days; in the PE microplastics treatment, only ∼10.9%. Also, oxygen consumption rate decreased with exposure	Lu and Liu (2021)
12	*Oreochromis niloticus*	Polystyrene microplastics	Polystyrene microplastics at doses of 0, 0.01, 0.1, 1 mg per 0.75 g feed	Growth results: Absolute weight growth, specific growth rate (SGR), and feed conversion ratio (FCR) were significantly affected by PS-MP dose; absolute length growth was *not* significantly different among treatments	Roch et al. (2022)
13	Nile tilapia (juveniles) in biofloc system	Polystyrene MPs	Polystyrene MPs (32–40 µm), added to biofloc; exposures 0, ∼80 items/L (∼30 μg/L), 800 items/L (∼300 μg/L) for 28 days	No significant effect on body weight gain over 28 d in this BFT setting, though liver accumulation, altered body composition (protein/lipid decrease), increased HSI and antioxidant enzyme responses were observed: indicating sublethal effects that may affect longer-term growth	Hu et al. (2022)

However, some organisms may display resistance to stressors caused by MNPs exposure, and since microplastics can be egested, their cumulative effects may sometimes be reduced. Nevertheless, aquatic organisms often ingest MNPs mistakenly, considering them as food particles, thereby introducing them into the food web and resulting in cumulative impacts through biomagnification (Au et al., 2017). Consequently, MNPs not only disrupt aquatic ecosystems but also threaten food safety, since fish are consumed by humans (Ghosh, 2018). After ingestion, MNPs accumulate in the body, migrate into cells, enter the circulatory and lymphatic systems, and distribute to different organs. This causes both functional and structural degradation of the GIT, ultimately impairing fish growth and nutritional status (Wang T et al., 2020). A recent study by Rashid et al. (2025a) revealed that dietary polystyrene microplastics (PS-MPs) significantly impaired growth in *Labeo rohita*. Fish receiving the highest inclusion level (2.5%) recorded the lowest final weight gain (21.55 g), while intermediate levels (0.5%–2%) produced a gradual decline in growth, accompanied by increased FCR and reduced SGR. Severe growth impairment at 2.5% PS-MPs coincided with the highest FCR (2.45) and lowest SGR (0.54). The ingestion of MPs caused gastrointestinal damage and induced oxidative stress through ROS overproduction, activating c-Jun N-terminal kinase (JNK) and p38 MAPK pathways. This stress-mediated response inhibited IGF-1 signaling and its downstream mTOR pathway, leading to reduced protein synthesis and growth retardation (Kadac-Czapska et al., 2024).

### 4.2 Effect of micro/nanoplastics on oxidative stress of the fish

The generation of reactive oxygen species (ROS) following micro/nanoplastic (MNPs) exposure triggers antioxidant defense responses, characterized by increased activities of catalase (CAT), superoxide dismutase (SOD), glutathione peroxidase (GPx), and glutathione-S-transferase (GST), along with elevated levels of oxidative damage indicators such as lipid peroxides (LPO) and protein carbonyls. The detailed results are presented in Table 2. Excessive ROS generation, however, can surpass the physiological tolerance limits and subsequently reduce antioxidant enzyme activity (Mo et al., 2021). Moreover, the accumulation of lipid peroxidation by-products, such as malondialdehyde (MDA), in body tissues may inhibit antioxidative processes (Shen et al., 2019). High accumulation of MNPs in the gastrointestinal tract has been associated with elevated oxidative stress markers, including increased activities of SOD, CAT, GPx, and GST (Mannering et al., 2025). Although the exposure pattern of MNPs is complex, it clearly alters redox homeostasis in fish. MNPs toxicity is recognized as a primary cause of oxidative stress, leading to disruption of redox equilibrium, excessive ROS formation, and cellular damage (Kim et al., 2021). The resulting oxidative stress can cause cellular injury, lipid peroxidation, DNA damage, apoptosis, and protein denaturation (Hoseinifar et al., 2021). When ROS levels rise rapidly, fish exposed to MNPs tend to lose redox balance and redirect energy toward the synthesis of compounds and antioxidant enzymes that enhance antioxidant defense. Fish possess two major defense mechanisms against oxidative stress: an enzymatic pathway involving antioxidant enzymes (e.g., SOD, CAT, GPx, GST) and a non-enzymatic pathway that relies on antioxidants such as vitamin C, glutathione, thioredoxin, and vitamin E (Hoseinifar et al., 2021).

**TABLE 2:** Effect of micro/nanoplastics on oxidative of the fish.

S. no	Species	Type of micro/Nanoplastics	Size/Dose	Response	References
1	*Oreochromis niloticus*	Polystyrene (PS)	Polystyrene (PS) microspheres, several sizes (μm), sub-lethal concentrations	PS-MPs induced tissue-specific oxidative stress and inflammation; antioxidant responses were altered (up- or downregulated depending on tissue. ROS, altered SOD, IL-1β, TNF-α pro-inflammatory markers in gill, liver, and brain enhanced	Zheng et al. (2023)
2	*Sparus aurata*	MPs-enriched diets; virgin MPs and weathered MPs	MPs-enriched diets; virgin MPs and weathered MPs for 21 days	Weathered MPs elevated primary antioxidant enzyme activities in brain evidence of oxidative challenge. Enhanced SOD and CAT activities in brain tissue	Rios-Fuster et al. (2021)
3	*Oreochromis niloticus*	PS microplastics + copper (co-exposure)	0.1 μm PS-MPs+ Cu^2+^ levels: 0, 1, 2.5, 5, 10, 15 and 20 mg/L	MPs can exacerbate oxidative damage when present with co-contaminants (metals). Reduced total antioxidant capacity, altered CAT/SOD/GPx; Reduced MDA (lipid peroxidation)	Zhang et al. (2022)
4	*Seriola lalandi*	red polyethylene spherical beads at a density of 1.07 g/cc and a size range of 300–335 μm	Biofouled PE microplastics (300–335 µm)	Biofouled plastics can modulate oxidative biomarkers and metabolic stress in juveniles. Changes in metabolic rate; altered antioxidant biomarkers (SOD/CAT) and biomarkers of oxidative stress	Kelly et al. (2024)
5	*Carassius auratus*	PS-NPs	PS-NPs at 0, 1, 10, and 100 mg/kg	PS-NPs induce oxidative stress and perturb physiological parameters. Enhanced markers of oxidative stress (MDA, ROS), altered SOD/GPx activities; blood biochemistry changes	Azarm-Karnagh et al. (2025)
6	*Danio rerio*	MPs + Cu	MPs (2 mg/L) + Cu (25 μg/L) for 30 days	Co-exposure causes oxidative stress in gills; antioxidant enzyme activity is suppressed when both stressors present. ↑ ROS, ↑ lipid peroxidation (LPO/MDA), inhibited CAT and GPx activities	Santos et al. (2022)
7	*Pseudobagrus fulvidraco*	Polyamide microplastics (PA-MPs)	Polyamide microplastics (PA-MPs), varied conc. (10–10,000 mg/L) for 96 h	PA-MP exposure leads to activation of some antioxidant defenses (SOD, CAT), but also stress (oxidative damage) and altered blood/metabolic biomarkers. ↑ SOD and CAT at many exposure levels; ↓ GST; signs of lipid peroxidation, changes in plasma biochemistries	Jo et al. (2025)
8	*Coregonus peled Gmelin*	Polystyrene (PS) microspheres (size	PS microspheres with a size of 2.0 ± 0.2 µm	Antioxidant defense enzymes are modulated; sometimes increased (activation) then possibly overwhelmed. Changes in CAT, GST, GR, GPx activities in response to PS microplastics; direction of change depends on concentration and exposure time	Frank et al. (2023)
9	*Channa argus*	Polystyrene microplastics	Polystyrene microplastics (PS-MPs), at different ng/L exposure (500–2000 ng/L) for 4 weeks	MNPs exposure damaged antioxidant defense, increased oxidative damage across multiple organs; also triggered other stress pathways (inflammation, apoptosis, autophagy). ↑ ROS levels; ↑ MDA; ↓ SOD, CAT, GSH, GSH-PX enzymes; downregulation of genes related to antioxidant function (sod, cat, gsh-px etc.)	Jiao et al. (2025)
10	*Scophthalmus maximus*	MPs	MPs (sizes 50–200 µm), fibers and fragments; observed *in situ* (natural environment)	Even in wild fish, environmental microplastic burden associates with oxidative stress markers in multiple tissues; suggests possible human intake via muscle. Changes in SOD, CAT, GPx, PON, AR, MPO; elevated MDA; oxidative damage in brain, liver, gill, muscle tissues	Köktürk et al. (2024)
11	*Danio rerio*	Polystyrene NPs	Polystyrene NPs of different sizes (50 nm, 1 µm)	Smaller NPs (50 nm) distribute more widely; oxidative stress and suppressed antioxidant gene expression led to higher vulnerability, especially under further stress. ↑ ROS; decreased expression of catalase gene; altered antioxidant gene expression; immune cell migration; cellular oxidative damage markers	Cao et al. (2024)
12	*Cyprinus carpio*	Polypropylene (PP) microplastics (diet + water exposure), various concentrations	Polypropylene (PP) microplastics (diet + water exposure), various concentrations	PP microplastic exposure causes oxidative stress in gills; antioxidant defenses are affected (some up, some down) depending on exposure concentration/time. ↑ Malondialdehyde (MDA); ↑ glutathione (GSH); ↓ catalase (CAT); changes in TPC (total protein content)	Yedier et al. (2023)
13	*Oncorhynchus mykiss*	Fluorescent MP microbeads (1–5 μm), 50 mg/kg feed; combined with microencapsulated antioxidant (astaxanthin) in diet	Fluorescent MP microbeads (1–5 μm), 50 mg/kg feed; combined with microencapsulated antioxidant (astaxanthin) in diet for 60 days	Dietary antioxidant mitigated MP-induced oxidative stress; also reduced MP uptake and tissue translocation. Upregulation of *sod1, sod2, cat* gene expression; reduced oxidative stress markers when antioxidant was supplemented	Sener et al. (2025)
14	*Barbus sharpeyi*	PE-MP	PE-MP concentrations of 0, 200, 400, 800, and 1,600 mg/L for 21 days	PE microplastics cause oxidative stress and liver damage in Barbus sharpeyi. Changes in antioxidant biomarkers (CAT, SOD, etc.), elevated oxidative damage indicators; biochemical parameter disturbance in liver (hepatotoxicity)	Beitsayah et al. (2025)
15	*Oryzias latipes*	Polystyrene nanoplastics	Polystyrene nanoplastics; concentration of 0, 10, 10^4^, and 10^6^ particles/L	Long-term NP exposure harms gonad function; oxidative stress is part of the mechanism. ↓ SOD, CAT, GPx, lysozyme; changes in MDA; gonadal histology disruptions (reduced spermatogenesis/oogenesis)	Zhou et al. (2023)
16	*Pomacentrus amboinensis*	Microplastics with DEHP; ingestion exposure; varying number of particles; relatively low doses	2 days + predator exposure over 22 h mesocosm; fairly acute/subacute	Even short exposures that do not kill fish show oxidative stress; may have downstream costs (growth, energy allocation) though not immediate mortality. Biomarkers of oxidative damage and antioxidant metabolism elevated; damage correlated with particle load; antioxidant defense increased	Mannering et al. (2025)

Superoxide dismutase (SOD) acts as the primary antioxidant enzyme by converting superoxide radicals into hydrogen peroxide (H_2_O_2_), which is subsequently degraded into water and oxygen by catalase (CAT). Glutathione S-transferase (GST) plays a dual role in transporting hydrophobic molecules and detoxifying cytotoxic compounds, thereby protecting cells from oxidative injury. As part of the phase II detoxification system, GST conjugates glutathione with xenobiotics, including microplastics, reducing their toxicity (Kim et al., 2021). Oxidative stress increases energy demand in fish and may suppress antioxidant enzyme activities over time (Hamed et al., 2020). MNPs exposure interferes with ROS-related pathways, resulting in altered antioxidant responses (Wang et al., 2019). Glutathione (GSH) is central to maintaining redox balance during MNP exposure, while glutathione peroxidase (GPx) detoxifies H_2_O_2_ by converting it to water and oxidizing GSH to GSSG (Huang et al., 2020). Glutathione reductase (GR) subsequently regenerates GSH from GSSG using NADPH (Solomando et al., 2020; Santos et al., 2020). Accordingly, GSH and its associated enzymes form the core of antioxidant defense against MNP-induced oxidative stress, making GSH a reliable biomarker of antioxidant status in fish (Kim et al., 2021). Malondialdehyde (MDA), a product of lipid peroxidation, serves as a sensitive indicator of oxidative lipid damage (Alomar et al., 2017). When ROS production overwhelms antioxidant capacity, severe cellular consequences ensue, including lipid peroxidation, DNA damage, apoptosis, and protein degradation (Hoseinifar et al., 2021), often driven by mitochondrial dysfunction.

### 4.3 Effect of micro/nanoplastics on reproduction of the fish

Micro/nanoplastics (MNPs) enter aquatic organisms primarily through ingestion from water and diet. After uptake, these particles accumulate in the gastrointestinal tract and metabolically active organs, where they interfere with reproductive physiology in fish (Wang et al., 2022). The reproductive toxicity of MNPs in fish is summarized in Table 3. Polystyrene (PS) microplastics, among the most extensively examined polymers, have been repeatedly shown to exert detrimental effects on reproductive function. Owing to its widespread use in packaging, laboratory materials, medical devices, and electronic components, PS represents a major environmental contaminant (Wagner et al., 2014; Abidin et al., 2022). Chronic MNPs exposure promotes oxidative stress in gonadal tissues by enhancing ROS production, thereby activating p53-mediated apoptotic pathways (Qiang and Cheng, 2021). In zebrafish, PS microplastics significantly impaired sperm motility without affecting testis weight, sperm density, or semen volume, while sperm kinematic indices (linear velocity (VSL), average path velocity (VAP), and curvilinear velocity (VCL)) remained unchanged (Zheng et al., 2024). In addition to reproductive impairment, PS exposure negatively influenced embryonic development, as evidenced by reduced heart rate and hatching success, increased apoptotic cell death in neural and somatic tissues, and a higher frequency of developmental deformities (Zheng et al., 2024).

**TABLE 3:** Effect of micro/nanoplastics on reproduction of the fish.

S. no	*Species*	Type of micro/Nanoplastics	Size/Dose	Response	References
1	*Danio rerio*	Polystyrene microplastics	Polystyrene microplastics (PS-MPs); ∼1–5 μm; waterborne exposures (chronic)	PS-MP exposure caused gonadal damage, disrupted oogenesis/spermatogenesis, reduced spawning and fecundity; histological lesions in gonads	Qiang and Cheng (2021)
2	*Danio rerio adult females*	Polystyrene nanoplastics	Polystyrene nanoplastics of (PS-NPs); nm scale; sub-chronic	PS-NPs disrupted female reproductive health and fertility via modulation of SIRT1 pathway; reduced oocyte maturation and lower fertility metrics	Gupta et al. (2023)
3	*Coregonus lavaretus*	Carboxyl-coated polystyrene nanospheres	50 nm carboxyl-coated polystyrene nanospheres; sperm pre-fertilization exposure (0/100/10,000 particles per spermatozoon)	Highest NP concentration reduced sperm motility; delayed hatching; offspring body mass reduced; swimming performance impaired	Yaripour et al. (2021)
4	*Oryzias melastigma*	Polystyrene microplastics	Polystyrene microplastics (PS-MPs) in water; various concentrations; exposure during early development	PS-MP exposure delayed hatching time; reduced hatching rate; altered heartbeat; upregulation of genes for immune response; metabolic and developmental pathways perturbed	Chen et al. (2020)
5	*Marine medaka (Oryzias melastigma) adults + progeny*	Polystyrene microplastics	∼10 μm PS microplastics; realistic environmental concentrations (2, 20, 200 μg/L); exposure duration ∼60 days	Exposure delayed gonad maturation; reduced female fecundity; decreased plasma estradiol and testosterone; parental exposure delayed incubation, reduced hatching rate, reduced heart rate and body length in offspring	Wang et al. (2019)
6	*Gasterosteus aculeatus*	Polyester fibers (PET)	Polyester fibers (PET), diameter ∼9.7 µm, length ∼245 μm; concentration ∼1 × 10^4^ fibers/L; fertilization *in vitro* + development	No significant effect: Fertilization ∼ control; hatching similar; low mortality and abnormality; heart rate unchanged	Bunge et al. (2021)
7	*Salmo trutta*	Microplastic particles	Microplastic particles of PS, PET, PE ∼3,000 μm; long-term exposure (113 days); environmental realistic concentrations in sediment (0.1% w/w)	No observed adverse effect on hatching success, larvae survival, growth, yolk sac absorption, or incubation period under those conditions	Jakubowska et al. (2020)
8	*Cyprinus carpio*	PVC microplastics	PVC microplastics in diet (food-borne), 10%, 20%, 30% (by diet ration) over 60 days	Gonadosomatic index (GSI); gonadal development; brain and gonad histology; sex hormone levels; expression of HPG axis genes; apoptosis gene expression	Liu et al. (2023)
9	*Oryzias melastigma*	Co-exposure: 17α-ethynylestradiol (an estrogenic endocrine disruptor) + polystyrene microplastics	Co-exposure: 17α-ethynylestradiol (an estrogenic endocrine disruptor) + polystyrene microplastics (various concentrations 2–200 μg/L); duration ∼28 days	Gonadosomatic index, hepatosomatic index; plasma E2 levels; E2/T ratio; transcription of estrogen biomarker genes (vitellogenin, choriogenin, estrogen receptors); histological damage to testes and liver	Wang et al. (2022)
10	*Poecilia reticulata*	Polystyrene nanoplastics	Polystyrene nanoplastics (PS-NPs), 50 μg/L; gestational exposure 30 days	Gestational PS-NP exposure reduced pregnancy rate and number of offspring; embryos accumulated PS-NPs with oxidative imbalance	Malafaia et al. (2022)
11	*Clarias gariepinus*	Styrofoam microplastics	Styrofoam microplastics (feed mixed) at 5%, 10%, 15% of feed; exposure 30 days	Significant reductions in GSI and GML at higher MP levels (15%); delayed or altered testis cell structure; increased vacuolation and possible apoptosis of gonadal cells at high MP feed percentage	Laily et al. (2023)
12	*Paramisgurnus dabryanus*	Polyethylene (PE) microplastics	Polyethylene (PE) microplastics; 1 mg/L and 10 mg/L; exposures 15 or 30 days in F0; looking also at F1	In F0: PE-MPs accumulate in gonads, cause pathological lesions, increase apoptosis, disturb HPG gene transcription and sex hormones, degrade semen quality. In F1: Embryos show increased mortality, malformation, decreased hatching rate, reduced body length	Xia et al. (2023)
13	*Oryzias latipes*	Polystyrene microplastics	Polystyrene microplastics (2-µm fluorescent PS-MPs), exposure ∼10^7^ particles/L for 3 weeks	High accumulation of PS-MPs in intestines; gene-expression changes in intestinal tissue; no significant effects on survival or reproduction over that 3-week exposure	Assas et al. (2020)

Several studies have demonstrated that exposure to MNPs disrupts multiple stages of the reproductive cycle in aquatic animals, including gametogenesis, gamete and oocyte quality, fertilization rate, egg production, and sperm swimming activity (Sussarellu et al., 2016). The gonadosomatic index (GSI), commonly used to assess reproductive health in fish, has also been shown to vary significantly under MNPs exposure across different species (Wang et al., 2019). In female guppies, MNPs exposure led to reduced pregnancy rates and decreased embryo counts (Malafaia et al., 2022). Similarly, zebrafish and medaka exposed to MNPs exhibited a decline in mature sperm and egg counts, which consequently reduced fertilization success and egg production (Cormier et al., 2021; González-Doncel et al., 2022). Additionally, MNPs exposure has been linked to oocyte degeneration and delayed hatching (Cormier et al., 2021), as well as reduced sperm motility (Yaripour et al., 2021).

Growing evidence suggests that micro/nanoplastics (MNPs) interfere with reproductive endocrinology in fish by altering the transcription of genes associated with the hypothalamic-pituitary-gonadal (HPG) axis (Liu et al., 2023). The HPG axis is central to the regulation of gonadal steroidogenesis and reproductive competence in teleost fishes (Huang et al., 2022). MNPs exposure has been shown to dysregulate hormone-responsive genes in the brain, including estrogen receptors (ER-α and ER-β), gonadotropin-α (GTHα), gonadotropin-releasing hormone (mGnRH), and androgen receptor (AR-α) (Wang et al., 2022; Yan et al., 2020). At the pituitary level, MNPs induced endocrine disruption is reflected by significant changes in the expression of follicle-stimulating hormone β (FSHβ), luteinizing hormone β (LHβ), and gonadotropin-releasing hormone receptor (GnRHR). In the gonads, altered expression of FSHR and LHR further indicates impaired gonadotropin signaling (Karami et al., 2016; Bhagat et al., 2021). Additionally, MNPs exposure affects genes involved in steroid hormone biosynthesis and metabolism, including StAR, multiple hydroxysteroid dehydrogenases, and cytochrome P450 enzymes (CYP11a, CYP17). Disruption of estradiol synthesis genes (CYP19a and CYP19b) and gonadal differentiation regulators (dmrt1 and sox9) highlights the potential of MNPs to interfere with sex differentiation and reproductive development (DiBona et al., 2022; Fernandez-Míguez et al., 2023).

Interestingly, the size of microplastics (MPs) in aquatic environments such as rivers and oceans ranges from 1 μm to 5 mm. Through microbial degradation, photooxidation, and thermal oxidation, these particles can further fragment into smaller units known as nanoplastics (NPs) (Wang et al., 2019). Exposure to micro- and nanoplastics (MNPs) has been shown to affect the transcription of key regulatory genes such as gthα, lhβ, and shβ in the reproductive axis of both male and female fish (Wang et al., 2021). Moreover, dietary incorporation of NPs was reported to influence the physiological condition of offspring. Additionally, nanoparticles initially accumulated in the yolk sac and were subsequently transferred to the eggs and hatching juveniles, with eventual distribution to the intestine, pancreas, and liver tissues (Pitt et al., 2018). These findings suggest that NPs can be maternally transferred from parents to offspring, with additional observations of delayed swim bladder development. Interestingly, MPs alone did not significantly alter sperm histopathology; however, exposure to NPs resulted in a slight reduction in mature sperm counts. In female fish, nanoplastics (NPs) exposure disrupted ovarian oocyte development across stages including perinuclear oocytes (POs), cortical vesicle oocytes (COs), early vitellogenic oocytes (EVs), and late vitellogenic oocytes (LVs) (He et al., 2021). Furthermore, NPs can induce oxidative stress in developing follicles, disrupt the expression of critical regulatory genes, and impair follicular growth and function. For instance, downregulation of CYP1A1 expression can suppress estradiol (E2) synthesis, ultimately impairing follicular development (Britt et al., 2004; Gupta et al., 2010). A study on European whitefish (*Coregonus lavaretus*) exposed to PS-NPs reported enhanced sperm linearity and an increased proportion of immotile sperm (Yaripour et al., 2021). While fertilization success was not significantly reduced, exposed embryos hatched earlier than controls. Such changes may be attributed to oxidative or epigenetic modifications in sperm, affecting post-fertilization development and stress tolerance. Collectively, the results demonstrate that PS-NPs can penetrate biological barriers, accumulate in germ cells, disrupt apoptosis and meiosis, impair sperm motility, and alter embryonic developmental timing. Such cellular-level disruptions represent a critical pathway by which PS-NPs compromise male reproductive fitness and potentially threaten population-level reproductive success in aquatic organisms. Furthermore, evidence indicates that parental exposure to MNPs disrupts physiological development, alters embryogenesis, and promotes the transgenerational transfer of toxicity, thereby jeopardizing offspring viability and ecosystem sustainability (Cheng et al., 2025; Liao et al., 2023). In addition, MNPs exposure has been associated with embryonic morphological abnormalities, including spinal curvature, pericardial cyst formation, and growth retardation.

### 4.4 Effect of micro/nanoplastics on endocrine system of the fish

Exposure to micro/nanoplastics (MNPs) has been shown to disrupt the endocrine system, leading to dysregulation of reproductive hormones in fish. Sex hormones play a vital role in maintaining endocrine homeostasis; therefore, any alteration in these hormones can disturb the entire system (Lin et al., 2023). Testosterone (T) and estradiol (E2) are key steroid hormones regulating sexual development, differentiation, and reproduction in fish. Estradiol primarily governs ovarian development, whereas testosterone is essential for spermatogenesis and sperm release (Zhang et al., 2016). Exposure to micro/nanoplastics (MNPs) has been reported to disrupt gonadal structure and alter the secretion of T and E2, thereby impairing reproductive endocrine function (Wang et al., 2022). The effects of micro/nanoplastics on endocrine disruption in fish are summarized in Table 4. MNPs may also elicit sex-specific responses by modulating circulating hormone levels (Mallik et al., 2021). Vitellogenin (VTG) and choriogenin (CHG), which are induced by estradiol, are critical for yolk and egg envelope formation in female fish (Matozzo et al., 2008). Although VTG is typically absent in males, its induction serves as a sensitive biomarker of exposure to estrogenic contaminants (Sarasamma et al., 2020; Das et al., 2023). MNPs exposure has also been shown to suppress VTG secretion in a concentration-dependent manner, adversely affecting egg quality and embryonic survival (Huang et al., 2022). Furthermore, exposure to virgin microplastics disrupts follicular development and vitellogenesis in ovaries and causes structural disorganization of seminiferous tubules and spermatocytes in testes (Wang et al., 2019).

**TABLE 4:** Effect of micro/nanoplastics on endocrine disruption of the fish.

S. no	*Species*	Type of micro/Nanoplastics	Size/Dose	Response	References
1	*Oryzias melastigma,* adult male	Polystyrene microplastics (PS-MPs) co-exposed with 17α-ethinylestradiol	Polystyrene microplastics (PS-MPs) co-exposed with 17α-ethinylestradiol (EE_2_); 2–200 μg/L MPs; 1–10 ng/L EE_2_	MPs increased the estrogenic potency of EE_2_: Higher VTG, raised E2/T ratio, stronger ER and vitellogenin induction and worsened gonad/liver histopathology vs. EE_2_ alone	Wang et al. (2022)
2	*Danio rerio, a*dult female	Polystyrene microplastics	Polystyrene microplastics (PS-MPs), chronic/subchronic (various doses)	PS-MPs disrupted hepatic VTG metabolism, altered plasma sex hormones and HPG gene expression, and reduced fecundity	Mohammadzadeh et al. (2024)
3	*Danio rerio*	Polystyrene nanoplastics	Polystyrene nanoplastics (PS-NPs); environmental concentrations; chronic	PS-NPs altered sex hormones and VTG and modulated HPG axis gene expression (evidence of endocrine perturbation linked to oxidative stress)	Sun et al. (2024)
4	*Oryzias latipes*	PET/environmental MPs	PET/environmental MPs long-term exposures (field-realistic)	At environmentally realistic concentrations PET MPs had negligible additive endocrine-disrupting activity in medaka — highlights importance of dose and particle chemistry	Nam et al. (2024)
5	*Marine medaka/Zebrafish (embryos and larvae)*	Aged plastic	Aged plastic leachates and weathered plastics (chemical leachates)	Leachates from weathered plastics induced endocrine responses in embryos showing that additives/Leachables from plastics can be endocrine active	Qiu et al. (2023)
6	*Medaka/Zebrafish*	PS-MPs/PS-NPs that sorb EDCs (BPA, PAHs, EE_2_	​	MPs act as vectors for sorbed EDCs increasing bioavailability of endocrine disruptors in fish and amplifying endocrine endpoints	Rochman et al. (2013)
7	*Oryzias melastigma*	Synthetic microfibers (polyester) ingestion	One week after exposure to two different concentrations of microfibers (500 and 1,000 fibers/L	Microfiber ingestion caused short-term HPG axis disruption in males, interfering with normal maturation and steroid profiles	Kim et al. (2023)
8	*Zebrafish (long-term parental exposure studies)*	PS-MPs/PS-NPs parental exposures	Polystyrene microplastics (PS-MPs), 1 μm and 3 µm respectively, at 0.01, 0.1, 1.0 and 10.0 mgL^-1^, and were monitored up to 72 h	Parental exposure often leads to altered parental sex hormones and VTG, and offspring may show endocrine-related changes (variable, dose dependent)	La Pa et al. (2024)
9	*Acanthopagrus Latus*	Dietary/waterborne polyethylene or polypropylene microplastics	Dietary/waterborne polyethylene or polypropylene microplastics (larvae/adults); co-exposure with antibiotics (florfenicol) or other chemicals in some studies	MPs alter biotransformation enzymes and oxidative stress markers; studies that measured hormonal biomarkers reported changes (some reductions/disruptions in hormonal indices) indicates potential endocrine disturbance in a euryhaline species used in brackish farming	Xu et al. (2023)
10	*Fundulus heteroclitus and Pagrus major*	Experimental uptake and gut retention of MPs (juveniles)	MPs similar in size (≥0.25 mm) and composition (polyethylene) to MPs detected in fish intestines	Juvenile mummichogs ingest and retain MPs supports exposure pathway needed for endocrine disruption (even if endocrine endpoints not measured in that specific study)	Ohkubo et al. (2020)

Micro/nanoplastics (MNPs) have been reported to reduce testosterone and 17β-estradiol levels by downregulating the activity of hypothalamic-pituitary-gonadal (HPG) axis transcriptional factors in the brain and ovary of fish. Such disruption initiates a downstream cascade affecting hepatic vitellogenesis and oocyte development, which eventually postpones ovarian maturation. In contrast, males exhibited upregulated HPG-related transcriptional regulators and higher circulating sex hormone levels, indicating clear sex-dependent effects of MNPs exposure. Moreover, exposure to virgin microplastics (MPs) adversely affects gonadal development in tilapia through reduced sex hormone production and altered HPG axis signaling pathways. Vitellogenin (Vg) and choriogenin (Chg), two liver-derived proteins, are essential for ovarian development in fish (Hara et al., 2016). Several studies have demonstrated that acute or chronic exposure to virgin MPs significantly disrupts the production of these proteins and their associated transcriptional regulators, leading to compromised gamete quality (Sarasamma et al., 2020; Wang et al., 2019). Furthermore, virgin microplastic exposure has been linked to early maturation and spawning, accompanied by reduced fecundity, decreased gonadosomatic index, lower fertilization rates, and impaired gamete quality (Wang et al., 2021).

### 4.5 Effect of micro/nanoplastics on immunity of the fish

Micro/nanoplastics (MNPs) are small particles with strong adsorption properties that can cause harm through two major pathways: (i) physical and mechanical blockage, and (ii) chemical toxicity resulting from complex interactions (Abad Lopez et al., 2023). Owing to their toxic properties, MNPs accumulate in various fish tissues and compromise immune function by interfering with nutrient uptake and altering energy allocation (Hamed et al., 2019). The fish innate immune system comprises humoral components, physical barriers, natural killer cells, and phagocytic cells such as mononuclear macrophages and granulocytes, all of which are essential for host defense. Coordinated interactions among these components are necessary to elicit an effective adaptive immune response (Bhattacharya et al., 2010; Jeyavani et al., 2023). MNPs can infiltrate fish tissues, including lymphoid organs, causing damage to the circulatory and lymphatic systems. With prolonged exposure, they accumulate in secondary organs and enter cells and intercellular spaces through endocytosis and pericellular infiltration, ultimately impairing cellular integrity and activating immune responses (Yuan et al., 2022; Ma C et al., 2021).

MNPs disrupt biological circulation and induce immunotoxicity by transferring and accumulating among cells, tissues, and organs. They alter cellular metabolite activity and concentration, immune gene expression, immune cell function, and apoptosis, ultimately disturbing the metabolism of key immune organs such as the intestine and liver. Within the gastrointestinal tract (GIT), MNPs accumulate and disrupt gut microbiota, as well as secretory, intestinal, and immune cells that are essential for nutrient absorption, endocrine regulation, immune responses, and barrier functions (Weichselbaum and Klein, 2018; Evariste et al., 2019). The intestinal immune system plays an essential role in host defense by preventing damage from pathogens and toxins, while simultaneously managing immune responses to antigens originating from resident microbiota and dietary inputs. This functional balance is maintained by immune cells such as myeloid cells, innate lymphoid cells, and T cells distributed throughout the gut lamina propria and mesenteric lymph nodes, which are central to mucosal immunity (Kabat et al., 2014). MNPs exposure damages the intestinal system, leading to dysregulation of immune-related genes such as IL-10, IL-1β, and interferons (Bhagat et al., 2020; Cocci et al., 2022). Gu et al. (2020) reported that exposure to polystyrene microplastics (PS-MPs) of different sizes (100 nm, 5 μm, and 200 μm) for 21 days in zebrafish caused intestinal immune cell dysfunction, including effects on phagosome activity and immune system regulation, along with an increase in pathogenic bacteria abundance. Similarly, MPs reduced the abundance of actinomycetes in the zebrafish intestine, a group of microorganisms critical for antibiotic synthesis, thereby weakening intestinal barrier functions and increasing susceptibility to immune challenges (Qiao et al., 2019).

MNPs exposure alters immune cell function in the fish kidney, particularly affecting macrophages, leukocytes, and neutrophils. Neutrophils play a pivotal role in early antibacterial defense against invading foreign substances (Havixbeck and Barreda, 2015) and are recognized as sensitive biomarkers of organismal and population health (Smith and Lumsden, 1983). Polystyrene and polycarbonate nanoplastics activate neutrophil responses, including degranulation, phagocytosis, and neutrophil extracellular trap formation, alongside mild oxidative stress induction. *In vitro* neutrophil assays showed that PS nanoparticles (41 nm; 0.025–0.2 μg/μL) dose-dependently increased myeloperoxidase activity and stimulated extracellular tartrate-resistant acid phosphatase (TRAP) release in *Pimephales promelas* neutrophils (Greven et al., 2016).

In fish larvae, neutrophils are the earliest phagocytic cells recruited to sites of inflammation and function as primary scavengers, followed by macrophages that clear cellular debris and apoptotic cells (Iribarne, 2021; Kumar et al., 2024; Parkin and Cohen, 2001). Macrophages play a pivotal role in host defense through encapsulation and phagocytosis, and their activation is commonly assessed using acid phosphatase (ACP) and thioflavin T (ThT) assays. Extended exposure to microplastics (MPs) can ultimately lead to macrophage apoptosis (Bon et al., 2025; Young et al., 2020). Cheng et al. (2022) demonstrated that exposure to 100 nm and 50 nm nanoparticles increased neutrophil abundance while reducing macrophage numbers in the abdominal region of juvenile zebrafish. Furthermore, smaller nanoparticles more effectively stimulated neutrophil activation in zebrafish larvae, leading to enhanced hepatic inflammation, whereas larger nanoparticles induced higher levels of macrophage apoptosis, likely mediated by increased endoplasmic reticulum stress (Cheng et al., 2022; Zhao et al., 2024). In addition, nanoparticles significantly promoted the polarization of primordial macrophages toward the M1 phenotype in zebrafish embryos, resulting in elevated production of pro-inflammatory cytokines, including *IL-6* and *IL-1β* (Li et al., 2025; Martin et al., 2023). The details have been mentioned in the Table 5.

**TABLE 5:** Effect of micro/nanoplastics on immunity of the fish.

S. no	*Species*	Type of micro/Nanoplastics	Size/Dose	Response	References
1	*Atlantic salmon*	PE and PS microplastics	PE and PS microplastics, 1–5 μm; 0.05–50 mg L^-1^ for 1–72 h	Immune cells can phagocytose MPs (even at low conc.); phagocytic uptake differed by polymer (PE more readily taken up than PS) and tissue; low % of cells phagocytosed particles, but particles accumulated	Abihssira-García et al. (2020)
2	*Sparus aurata* and *Dicentrarchus labrax*	Virgin MPs such as PVC, PE	Virgin MPs (PVC, PE; with 40–150 μm size	Few immediate effects on cell viability, but oxidative stress and altered innate activities reported in continuing exposures; polymer type mattered	Espinosa et al. (2018)
3	*Sparus aurata*	PVC or PE	PVC or PE microparticles in diet. (100 or 500 mg of virgin PVC-MPs or PE-MPs per kg diet)	Dietary MPs produced mild immunosuppression and oxidative stress; longer exposures likely produce more severe immune impairment	Espinosa et al. (2019)
4	*Dicentrarchus labrax*	Functionalized polystyrene nanoplastics (PS-NPs)	Polystyrene nanoplastics (PS-NP; 50 nm) functionalized with carboxyl (PS-COOH) or and amine (PS-NH_2_)	Pre-exposure to functionalized NPs reduced antiviral immune responses and increased susceptibility to nodavirus (higher viral replication/worse outcomes)	Gonzalez-Fernandez and Cuesta (2022)
5	*Oryzias melastigma*	Polystyrene MPs	Polystyrene MPs with a diameter of 6.0 µm	PS-MP exposure altered immune gene profiles, increased ROS and modified phagocytic responses effect depended on concentration and duration	Chen J et al. (2022)
6	Fish and other models	MPs/NPs ± co-contaminants	MPs/NPs ± co-contaminants with different size and concentration	MPs/NPs produce immunotoxic effects in fish, often via oxidative stress and pro-inflammatory signaling; co-exposure with pollutants frequently exacerbates immune disruption	Li et al. (2024)
7	*Menidia beryllina*	Polylactic acid (PLA), cryo-milled tire particles (TP), and polyester microfibers (MF)	Polylactic acid (PLA), cryo-milled tire particles (TP), and polyester microfibers (MF). Fibers made up 80% of the particle collected in a study of the San Francisco Bay, followed by fragments at 17%	Effects are highly variable between studies; particle size, shape, polymer, functionalization, dose, and species drive heterogeneity; small NPs more consistently linked to immune modulation	Hutton et al. (2024)
8	*Danio rerio*	Polystyrene NPs and MPs via water; accumulation assessed in embryos and intestines; exposure before infection with *Edwardsiella piscicida*	Polystyrene NPs (100 nm) and MPs (5 µm) via water; accumulation assessed in embryos and intestines; exposure before infection with *Edwardsiella piscicida*	Both size classes (NPs and MPs) accumulate; cause oxidative stress; kidney damage (ER stress by NPs; lipid accumulation by MPs); suppression of innate immune signaling (C-type lectin, cytosolic DNA sensing); *il1b* downregulated; increased susceptibility to infection	Yang et al. (2023)
9	*Pimephales promelas*	Polystyrene nanoplastics	Polystyrene nanoplastics, exposure via ingestion and intraperitoneal injection; 48 h exposure	Nanoplastics were found in liver/kidney; observed significant downregulation of *ncf*, *mst1*, *c3*, affecting neutrophils/macrophages/complement system; route of exposure mattered	Elizalde-Velázquez et al. (2020)
10	*Danio rerio*	PS nanoplastics and micron PS particles	PS nanoplastics (50 and 100 nm) and micron PS particles; water exposure; dose- and size-dependent; several days in larval stage	NPs (especially smaller) induced greater neutrophil aggregation; macrophage apoptosis; raised *fabp10a* expression in larval liver; increased ROS; metabolic pathways altered	Cheng et al. (2022)
11	*Channa argus*	Polystyrene MPs (microplastics)	Polystyrene MPs (microplastics), doses: 500, 1,000, 2000 ng/L over 4 weeks in diet (or ambient) exposure	Exposure increased ROS and MDA, reduced antioxidant enzyme activity; immune parameters (C3, C4, LYS, IgM) altered (generally depressed) in organs; pathological changes in immune and barrier tissues	Jiao et al. (2025)
13	*Danio rerio*	Polystyrene MPs and NPs	Fluorescent or non-fluorescent NPs at levels of 100 μg/mL (n-100 group) and 1,000 μg/mL	PS-MNPs cause ROS production → NF-κB activation → increased pro-inflammatory cytokines, apoptosis; ROS inhibition reduced inflammation	Pei et al. (2024)
14	*Sparus aurata*	Dietary polystyrene MPs	Dietary polystyrene MPs (1–20 µm); 21-day	Gut barrier disruption and altered mucosal innate markers after dietary MPs; indicates gut mucosal immune impairment even at sub-lethal levels	Del Piano et al. (2023)
15	*Danio rerio*	PS-NPs co-exposed with silver nanoparticles (AgNPs); water exposure	PS-NPs co-exposed with silver nanoparticles (AgNPs); water exposure	PS-NPs acted as carriers increasing bioavailability/toxicity of AgNPs, amplifying oxidative stress and immunotoxic endpoints (synergistic effect with co-contaminant)	Yan et al. (2023)
16	*Sparus aurata*	Microplastics (MP)	Long-term exposure (90 d) to MPs	Chronic exposure elevated oxidative stress and altered inflammatory biomarkers — evidence for systemic immune/inflammatory modulation with long exposure	Solomando et al. (2021)
17	*Cyprinus carpio*	Polystyrene microplastics	Polystyrene microplastics (8 µm), ∼1,000 ng/L in water; feeding/water exposure	MPs induce oxidative stress, activate innate inflammatory signaling (TLR2-MyD88-NF-κB), cause tissue injury in liver-like organ (“hepatopancreas”), activation of TLR2 signaling pathway (MyD88, TRAF6, NF-κB p65); increases in inflammatory molecules (TNF-α, IL-1β, iNOS, COX2); hepatopancreas damage. in carp	Cui et al. (2023)
18	*Oreochromis niloticus*	Co-exposure: Polystyrene microplastics (PS-MPs) + copper (Cu^2+^)	Co-exposure: Polystyrene microplastics (PS-MPs) + copper (Cu^2+^) in water; various doses	Combined exposure worsens damage: Co-exposure leads to higher metal accumulation, greater inflammation/immune perturbation, gut and tissue damage	Zhang et al. (2022)
19	*Pimephales promelas*	Polystyrene nanoplastics (PS-NPs)	Polystyrene nanoplastics (PS-NPs), exposure via ingestion and intraperitoneal injection; 48 h exposure	PS-NPs downregulate expression of genes related to neutrophil function, macrophages, complement; both exposure routes lead to immunotoxicity in immune tissues	Elizalde-Velázquez et al. (2020)
20	*Ctenopharyngodon idella*	Exposure to polystyrene microplastics	Exposure to polystyrene microplastics (different sizes, concentrations, durations), followed by depuration; also testing mitigation via nano-selenium	MPs cause size- and concentration-dependent immune/oxidative stress and tissue damage; nano-selenium supplementation mitigated many adverse immune/histological effects	Hao et al. (2023)
21	*Carassius carassius*	Co-exposure of microplastics + cadmium (5 mg/L)	Co-exposure of microplastics (1 mg/L) + cadmium (5 mg/L) for 96 h (liver), and microbiota observed over 21 days	Combined exposure increases hepatic accumulation and inflammation; immune compromise via oxidative stress and reduced antioxidant enzyme activity	Wei et al. (2023)
22	*Nile tilapia (GIFT strain)*	Chronic exposure to polystyrene MPs of different sizes (75 nm, 7.5 µm, 750 µm), combinations; 14 days	Chronic exposure to polystyrene MPs of different sizes (75 nm, 7.5 µm, 750 µm), combinations; 14 days	Significant immune and oxidative responses; size- and concentration-dependent effects: Some sizes more damaging; also combinations with algae modulated responses	Zheng et al. (2023)

### 4.6 Effect of micro/nanoplastics on hematological indices of the fish

The hematological parameters such as RBC and WBC, hematocrit, and hemoglobin are significantly affected by MNPs in fish. MNPs can penetrate the circulatory system, leading to metabolic disorders and severe effects such as endocrine disruption, oxidative stress, immune alterations, and changes in gene expression (Trivedi et al., 2026). Approximately 3%–4% of ingested MNPs are absorbed in the gut, where they affect the circulatory system, impair blood circulation, and trigger allergic reactions or localized inflammation due to their size (either micro or nano) (Thapliyal et al., 2025; Bhattacharyya et al., 2025). Furthermore, microplastics can bind with blood proteins such as albumin and globulin to form plastic–protein complexes, which may further interfere with blood circulation (Ghosh, 2025; Rajendran and Chandrasekaran, 2023; Yong et al., 2020). Exposure to MNPs has been associated with damage to RBC generation, suppression of hemoglobin biosynthesis, and impairment of erythropoiesis. Disruption of erythrocyte maturation, along with the development of macrocytic or microcytic anemia, indicates a decline in hematological status caused by microplastic exposure (Iheanacho and Odo, 2020). Consequently, microplastic exposure in fish reduces hemoglobin levels and synthesis, leading to impaired tissue oxygenation and increased toxicity (Kim et al., 2021). Once absorbed, microplastics can diffuse across cellular membranes, enter the circulation, and bioaccumulate in the body. Therefore, hematological profiling can serve as a sensitive biomarker for microplastic exposure in fish (Scanes et al., 2019).

Exposure to microplastics significantly reduced hematological parameters such as hemoglobin (Hb), RBC count, and hematocrit values (Lee et al., 2025). Interestingly, the results also revealed that smaller MNPs caused greater reductions in these blood parameters even at lower concentrations, suggesting that smaller particle sizes are associated with higher sensitivity. The findings further indicate that MPs can enter the circulatory system and physically interact with blood cells, as evidenced by their accumulation in the liver. Such mechanical effects may damage cell membranes, ultimately leading to hemolysis and subsequent reductions in hematological indices. This is likely because only very small MPs can enter the bloodstream, and particle size distribution analysis showed that the MPs used in the experiment contained only a limited fraction of particles small enough to penetrate the fish’s circulation. Thus, even if the absolute number of MPs entering the bloodstream does not vary substantially, the lower LOEC observed for smaller MPs indicates that particle size is a key determinant, with smaller particles producing heightened sensitivity.

## 5 Effect micro/nanoplastics on rice

### 5.1 Effects of micro/nanoplastics on growth and development of plants

**FIGURE 2:**
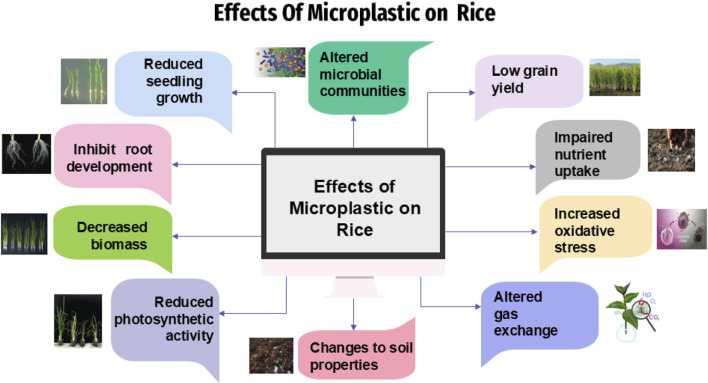
Effect of micro/nanoplastics (MNPs) on Rice.

Previous studies have shown that widespread use of polymer-based fertilizers, mulching films, biosolids and compost, wastewater irrigation and atmospheric deposition are the primary causes of microplastic pollution in agricultural farmlands (Kumar et al., 2020). Recent studies have revealed that microplastics of a size of 200 nm can be taken up by plants (Li et al., 2020). MPs are readily available substances and tend to accumulate in plants and have negative effects on growth and productivity (Kole et al., 2017; Taylor et al., 2020; Khalid et al., 2021). The impact of MPs on plant growth varies depending on the type of plant species and kind of plastic, potentially altering composition of plant community and primary productivity (Rillig et al., 2019). The effect of micro/nanoplastics (MNPs) on rice has been shown in Figure 2.

Previous studies have consistently reported an inverse relationship between plant growth and micro/nanoplastic (MNPs) concentration. Accumulating evidence indicates that MNPs induce oxidative stress, cytotoxicity, genotoxicity, and ecotoxicity in plants, while also disrupting essential physiological processes such as photosynthesis and nutrient uptake. In addition, MNPs can alter gene expression, compromise plant defense systems, and act as vectors for heavy metals and pathogenic microorganisms, thereby exacerbating ecological and agricultural risks (Chaudhary et al., 2025). The detrimental effects of MNPs on plants include: (i) impaired nutrient uptake and transport due to blockage of cytoplasmic pathways and cell-to-cell communication, along with inhibition of photosynthesis; (ii) delayed or reduced seed germination resulting from restricted water absorption (Jia et al., 2023); (iii) alterations in root and shoot growth and development (Boots et al., 2019); and (iv) broader ecotoxicological and genotoxic consequences (Jiang et al., 2019). Because plant roots are in direct contact with MPs in soil, root–microplastic interactions have received considerable research attention. Root exudates facilitate the adhesion of MPs to root surfaces, a process enhanced by the large surface area of MPs (Taylor et al., 2020), and some particles can be partially absorbed by root tissues (Zhou et al., 2021). Once internalized, MPs may be translocated to aerial plant parts, including stems and leaves, via transpirational pull (Lv et al., 2019). Li et al. (2020) demonstrated that plastic particles ranging from 0.2 to 2.0 μm could penetrate the root surfaces of *Triticum aestivum* L. and *Lactuca sativa* L. Similarly, scanning electron microscopy (SEM) studies have detected MPs in the stems and leaves of lettuce grown in MP-contaminated soils (Li et al., 2019). In leaves, the primary physical barrier against MP entry is the outer waxy cuticular layer (Arya et al., 2021). However, recent studies have shown that maize (*Zea mays* L.) leaves can absorb both non-labelled and fluorescently labelled plastic particles through stomatal openings. Following uptake, these particles are transported to vascular tissues via the apoplastic pathway and accumulate in trichomes. Such mechanisms have been elucidated using advanced analytical approaches, including hyperspectral imaging, confocal microscopy, and laser ablation–inductively coupled plasma mass spectrometry. Furthermore, numerous studies have demonstrated that micro- and nanoplastics (MNPs) adversely affect the growth and development of rice and wheat during both vegetative and reproductive stages (Chen et al., 2023; Qi et al., 2018). Overall, MPs can influence multiple stages of the plant life cycle and disrupt ecosystem functioning through diverse pathways (Khalid et al., 2021). Despite growing research efforts, significant gaps remain in our understanding of plant–microplastic interactions. A summary of the effects of micro- and nanoplastics on the physiological attributes of rice is presented in Table 6.

**TABLE 6:** Effects of micro/nanoplastics on physiological attributes of Rice.

S. no	Physiological attributes	Effects	References
1	A chlorophyll concentration (for PET)	Decrease in chlorophyll concentration 20% for chi a, 28% for chl b, and 18% for total chl	Iswahyudi et al. (2024)
	B. Chlorophyll concentration (for PP)	Decrease in chl concentration compared to pet exposure, with an average decrease of 15% for chl a, 21% for chl b, and 11% for total chl	Iswahyudi et al. (2024)
	C. Chlorophyll concentration (for PS)	Decrease of 43% for chl a, 41% for chl b, and 40% for total chl	Iswahyudi et al. (2024)
	D. Chlorophyll concentration (for As + NPs)	Nps + As reduced chlorophyll a by 38%–45% and chlorophyll b by 42%–53%	Mamathaxim et al. (2023)
2	Stomatal conductance	Declined under all MP treatments • PS-MPs at D1 (0.5 mg/L) → −13.84% • PVC-MPs at D4 (0.5 mg/L) → −19.57% • Severe decline at D6 (3.0 mg/L PVC) → −48.65%	Ma Y et al. (2022)
		Stomatal conductance (Gs) 3.40%–67.36%	Li et al., 2023
3	Chlorophyll content (SPAD)	PE reduced SPAD values (chlorophyll proxy), especially when combined with znonps	Tan et al., 2022
		Reduced consistently • PS-MPs at D1 → −15.56% • PVC-MPs at D4 → −24.76% • Maximum decline at D6 (PVC 3 mg/L) → −49.88%	Ma C et al. (2022)
4	Water use efficiency	Increased with higher MP doses • Maximum value recorded at D6 • But D3 (PS 3.0 mg/L) caused −35.92% reduction in WUE (anomalous dose effect)	Ma Y et al. (2022)
5	Internal CO_2_	Increased with increasing MP exposure • Maximum at D6 • D3 showed −35.67% reduction compared to control	Ma C et al. (2022)
		Intercellular CO_2_ (Ci) 3.66%–21.86%	Li et al., 2023
6	Transpiration rate	Highest in control (D0) • Gradual decline with increasing MP doses • Minimum values at D5 and D6	Ma Y et al. (2022)
		Transpiration rate (Tr) increased 10.79%–82.37%	Li et al., 2023
7	Photosynthetic rate	• Control (D0) = highest • PS-MPs (0.5, 1.5, 3.0 mg/L → D1, D2, D3) reduced photosynthesis by −11.78%, −18.72%, and −31.49% • PVC-MPs (0.5, 1.5, 3.0 mg/L → D4, D5, D6) reduced photosynthesis by −21.03%, −25.98%, and −43.81% • The strongest inhibition was at D6 (3.0 mg/L PVC)	Ma C et al. (2022)
		Photosynthetic rate (Pn) fell 6.36%–40.46%	Li et al., 2023
8	Nitrate and iron metabolism (for PS NPs)	OsMASD25, OsNAAT1, OsIRO2: Upregulated	Zhou et al. (2021)
9	Nitrate and iron metabolism (for PS NPs)	OsOFP2: Downregulated	Zhou et al. (2021)
10	Lignin biosynthesis (for PS NPs)	LAC6, LAC15, LAC19: Downregulated	Zhou et al. (2021)
11	Photosynthesis (for PE MPs)	OsPsb28, OsPsbO, OsPsbP, OsPsbQ, OsPsbR OsPsaH, OsPsaK, OsPetE, OsPetF, OsPetJ, OsLhcb1, OsLhcb2, OsLhcb4, OsLhcb6, OsPsbS, and OsPsa: Downregulated	Yang and Gao. (2022)
12	Ammonium and nitrate transporters (for PE MPs)	OsAMT1, OsAMT2, OsAMT3, and OsNRT2: Downregulated	Yang and Gao. (2022)
13	Nitrogen and glutamine metabolism (for PE MPs)	OsNR1, OsNR2, OsNiR1, and OsGS1: Downregulated	Yang and Gao. (2022)
14	Nitrogen and glutamine metabolism (for PE MPs)	OsNAR2.1, OsNRT2.2, and OsNRT2.3a: Upregulated	Yang and Gao. (2022)
15	Zn and Mn translocation (for PS-NPs)	OsZIP8, OsNRAMP3, and OsMTP8.1: Downregulated	Jiang et al. (2022)

### 5.2 Effect of micro/nanoplastics on seed germination of rice

The first phase in plant growth is seed germination and is extremely sensitive to various stress conditions (Wang and Tanveer, 2023) including MNPs stress. It has been recorded that the existence of MNPs in soil can delay the seed germination process and can also be transmitted from the roots to the stems and leaves, leading to interruption in plant growth (de Souza Machado et al., 2019). According to previous studies the presence of polyethylene MPs recorded reduction in seed germination rate and negatively affect the root and shoot characteristics of crops (Sahasa et al., 2023). Lentil seeds exposed to polyethylene MPs also demonstrated reduction in internal activity at the time of germination, which may be due to physical clogging of pores by MPs (De Silva et al., 2022).

### 5.3 Effect of micro/nanoplastics on seedling stage of rice

Micro/nanoplastics can adversely affect the growth of rice seedling depending on the type, size, concentration and exposure time. Dong et al., 2020 recorded that exposure to polystyrene and polytetrafluorethylene MNPs on rice seedlings cause reduction in biomass and also inhibited photosynthesis. In maize seedlings, PE-MNPs were also obstructed photosynthesis and inhibited arsenic accumulation (Sun H et al., 2023). Rice seedlings were exposed to nano-sized (80 nm) and micro-sized (1 µm) fluorescently labeled polystyrene (PS) microspheres for 14 and 40 days, respectively. After exposure periods, confocal laser scanning microscopy revealed the presence of both particle sizes in root, stem and leaves of rice seedlings (Liu et al., 2022b). Overall, MPs were observed to impair seedling growth and may negatively affect whole plant performance.

### 5.4 Effect of micro/nanoplastics on shoot and root growth of rice

Recent studies have shown that microplastics can markedly alter rice root development, leading to increased root diameter, surface area, volume, and even enhanced aboveground biomass (Hong et al., 2024). However, contrasting evidence suggests that MPs may also exert inhibitory effects on rice growth. PET microplastics have been reported to suppress root elongation, reduce shoot height, and lower fresh biomass. In addition, exposure to PET, PP, and PS microplastics decreased chlorophyll a, chlorophyll b, and total chlorophyll levels in rice leaves, reflecting compromised photosynthetic efficiency (Iswahyudi et al., 2024). Exposure to PS nanoplastics during early seedling development reduced root length but increased lateral root numbers (Zhou et al., 2021). Comparable growth inhibition has been reported in wheat, where 0.1 and 1.0 μm PS microplastics (50–100 mg L^-1^) significantly reduced root growth (Fang et al., 2024). Likewise, prolonged exposure of rice to polyethylene microplastics (200 and 500 mg L^-1^) resulted in decreased root length and dry weight after 21 days (Tang et al., 2022). These observations suggest that microplastic effects on shoot and root growth are highly variable and depend on polymer type, concentration, and exposure regime.

### 5.5 Effect of micro/nanoplastics on tillering stage of rice

The widespread use of plastic film mulching has caused substantial plastic residue accumulation in agricultural soils, which eventually degrades into microplastics (Yang and Gao, 2022). In this study, the effects of microplastics originating from PBAT-based biodegradable mulch films (BM) and polyethylene mulch films (PM) on rice growth were evaluated. Both BM- and PM-derived microplastics significantly suppressed plant development by reducing plant height and dry matter accumulation, while also impairing physiological performance, biochemical responses, and gene expression. Conversely, PAN and PET microplastics of 200 μm and 10 μm showed little influence on rice plant height and tiller formation (Chen J et al., 2022). These findings suggest that microplastics can disrupt rice growth during the tillering stage, with effects that vary across polymer types and particle sizes.

### 5.6 Effect of micro/nanoplastics on grain yield of rice

The influence of microplastics (MPs) on rice grain yield appears to be highly variable, depending on polymer type, concentration, and rice cultivar. Chen S et al. (2022) reported that the presence of MPs in paddy soil exerted a positive effect on rice grain yield. In contrast, polystyrene (PS) microplastics significantly reduced rice biomass and grain production, which was attributed to physiological toxicity and impaired root activity (Dong et al., 2021). Zhenghua et al. (2023) examined the effects of polyethylene (PE) microplastics on two rice cultivars, the conventional variety Nangeng 5,055 (NG) and the hybrid Jiafengyou 6 (JFY). Their findings showed that PE MPs did not affect grain yield in the conventional cultivar, whereas a significant yield reduction was observed in the hybrid rice, mainly due to a decline in grain number per panicle. Similarly, Wu et al. (2022) reported a reduction of approximately 10.6% in head rice yield of the Y900 cultivar under PS microplastic exposure, while the grain yield of XS123 increased by about 6.4%. Furthermore, Guo et al. (2023) documented substantial yield losses of 32.1% and 32.9% in rice exposed to low-density polyethylene (LDPE) and polylactic acid (PLA) microplastics, respectively, particularly when combined with heat wave stress. Overall, microplastics exert both stimulatory and inhibitory effects on rice grain yield; however, negative impacts predominate due to disruptions in plant growth and physiological processes. Notably, certain rice varieties exhibit tolerance or even modest yield enhancement under specific microplastic types or exposure levels.

## 6 Synergistic effect of micro/nanoplastics with other pollutants

According to previous research, micro/nanoplastics can strongly interact with contaminants and facilitate their adsorption onto MPs surface through both physical (e.g., electrostatic) and chemical interactions. These processes are often influenced by the hydrophobic nature if the contaminants (Fu et al., 2021). Various investigations have demonstrated that microplastics are capable of adsorbing wide range of contaminants, including polycyclic aromatic hydrocarbons (PAHs), polychlorinated biphenyls (PCBs), dioxi-like chemicals, polybrominated diphenyl ethers (PBDEs), toxic metals, pharmaceuticals as well as pesticides and herbicides (Hurley and Nizzetto, 2018; Du et al., 2020; Wang W et al., 2020). MPs can interact with heavy metals (HMs) like cadmium (Cd), lead (Pb), and arsenic (As), leading to either synergistic or antagonistic effects in plants. Recent research indicates that combined exposure to MPs and these contaminants can disturb multiple physiological and biochemical processes in plants, thereby elevating signs of toxicity (Kajal and Thakur, 2024).

For instance, Kaur et al. (2022) found that while individual exposure to polypropylene MPs and cadmium inhibited rice seedling germination rate, their combined application reduced overall toxicity in some cases. Both PP and Cd alone decreased root and stem length, fresh weight and antioxidant enzyme activities like catalase (CAT), peroxidase (POD) and superoxide dismutase (SOD), while dry weight remained unaffected. The combination of 13 μm PP + Cd showed an antagonistic effect on the growth of rice seedlings, while 6.5 μm PP + Cd had a synergistic effect, revealing that smaller PP MP particles (6.5 µm) had a more pronounced negative effect on rice seedlings as compared to larger particles (13 µm). Dong et al. (2021) observed that polystyrene and polytetrafluoroethylene (PTFE) microplastics reduced bioavailability of arsenic (As) in rice. However, further investigation is needed to better understand the interactions between MPs and pollutants.

Important points on synergistic effects of micro/nanoplastics with other Pollutants.

1. Carrier (Trojan horse) role for increasing pollutant bioavailability: Micro/nanoplastics (MNPs) readily adsorb other environmental contaminants, including heavy metals, pesticides, pharmaceuticals, and persistent organic pollutants (POPs), due to their high surface area and hydrophobic nature. This Trojan horse effect can increase the bio-accessibility and internal exposure of organisms to these co-contaminants, often amplifying toxic outcomes compared with exposure to each pollutant alone (Sun N et al., 2023).
2. Synergistic toxicity with pesticides and nano-pesticides: In experimental exposures, nanoplastics combined with nano-pesticides induced stronger oxidative stress (ROS and lipid peroxidation) and antioxidant system disruption in *Artemia salina* than either pollutant alone, demonstrating synergistic toxicity in invertebrates (Kamalakannan et al., 2024).
3. Enhanced toxic effects with heavy metals: Co-existence of MNPs and heavy metals such as cadmium (Cd) and lead (Pb) can increase the accumulation and toxicity of metals in plants and soil organisms, elevating oxidative damage and altering physiological processes more than individual exposures (Kajal and Thakur, 2024).
4. Synergistic impacts documented in meta-analyses: Meta-analysis of plant studies has shown that combined exposure to MNPs and heavy metals produces stronger phytotoxic effects than heavy metals alone, with physiological responses (e.g., oxidative stress) generally enhanced under co-exposure conditions (Wu et al., 2024).
5. Interactions with pesticides and insecticides: Studies combining microplastics with pesticides such as deltamethrin found that the presence of MPs at environmentally relevant concentrations significantly increased toxicity to aquatic invertebrates, reducing survival rates more than either pollutant individually (Bastante-Rabadan and Boltes, 2024).
6. Synergistic effects with antimicrobials: Co-exposure of microplastics and antimicrobial compounds (e.g., antibiotics) in both aquatic and terrestrial animals frequently leads to synergistic toxicity, including amplified oxidative stress, immune disruption, altered gene expression, metabolism dysfunction, and gut microbiota imbalance (Tang, 2025).
7. Synergistic interactions with organic chemical pollutants: Interactions between microplastics and organic contaminants (e.g., chlorpyrifos, BPA, pharmaceuticals) have been shown to enhance ecotoxicological effects including mortality, growth inhibition, and changes in biomarkers, indicating that MPs can exacerbate the adverse impacts of these chemicals on organisms (Menendez-Pedriza and Jaumot, 2020).
8. Greater prevalence in aquatic systems: In this study indicates that synergistic toxic effects between MNPs and co-pollutants are observed more frequently than antagonistic effects in aquatic environments, suggesting that combined exposures often pose higher ecological risk than individual pollutants (Bastante-Rabadan and Boltes, 2024).
9. Modulation by environmental and particle factors: The degree of synergistic interaction depends on several factors, including the size and aging of microplastics, environmental conditions (e.g., pH, salinity), and chemical characteristics of co-contaminants. Smaller, aged MPs tend to adsorb more pollutants and thus strongly influence combined toxicity (Bastante-Rabadan and Boltes, 2024).
10. Implications for risk assessment and ecosystem health: Because microplastics often co-occur with other pollutants in real environmental scenarios, their combined effects can lead to unexpected biological responses and increased toxicity risk, underscoring the need to consider pollutant mixtures in ecological risk assessments (Bhagat et al., 2021).

## 7 Conclusion and future direction

The present review comprehensively synthesizes current knowledge on emerging contaminants, particularly micro/nanoplastics (MNPs), and their impacts on two ecologically and economically important components of food systems such as fish and rice. The evidence clearly indicates that MNPs exposure adversely affects multiple biological endpoints in fish, including growth and development, immune function, antioxidant defense mechanisms, neurotransmission, endocrine regulation, reproductive performance, and metabolic homeostasis. Similarly, in rice, MNPs have been shown to impair seed germination, root and shoot growth, vegetative development, tillering, and ultimately grain yield, highlighting their potential threat to crop productivity and food security. By integrating findings across aquatic and agro-ecosystems, this review provides valuable insights for policymakers, researchers, and environmental regulatory agencies, supporting the development of effective monitoring strategies and control mechanisms for MNPs pollution across ecosystems. Despite significant progress, important research gaps remain. Future studies should focus on size-specific effects of micro/nanoplastics in aquatic animals and crop plants, as particle size plays a critical role in bioavailability and toxicity. Moreover, systematic evaluation of lethal and sub-lethal concentrations of different MNPs types is urgently needed, particularly in widely consumed fish species such as carps, catfishes, and commercially important marine fishes. Priority should also be given to assessing MNPs contamination in rivers, canals, reservoirs, and water bodies influenced by industrial activities. Addressing these gaps will be essential for accurate risk assessment and for formulating evidence-based policies aimed at mitigating the impacts of micro- and nanoplastics on ecosystems and human health.

## Glossary

MNPs: Micro/nanoplasctics
GIT: Gastrointestinal tract
PE: Polyethylene
PP: Polypropylene
PS: Polystyrene
LDPE: Low-density polyethylene
HDPE: High-density polyethylene
PVC: Polyvinyl chloride
PET: Polyethylene terephthalate
POM: polyoxymethylene
PBAT: polybutylene adipate terephthalate
PLA: Polylactic acid
SGR: Specific growth rate
FCR: Feed conversion ratio
ROS: Reactive oxygen species
JNK: c-Jun N-terminal kinase
IGF: Insulin-like growth factor-1
mTOR: Mechanistic target of rapamycin
MAPKs: Mitogen-activated protein kinases
CAT: Catalase
SOD: Superoxide dismutase
GPx: Glutathione peroxidase
GST: Glutathione-S-transferase
MDA: Malondialdehyde
H2O2: Hydrogen peroxide
GR: Glutathione reductase
VSL: Linear velocity
VAP: Average path velocity
VCL: Curvilinear velocity
ER: Estrogen receptors
GTHα: Gonadotropin-α
mGnRH: Gonadotropin-releasing hormone
AR: Androgen receptor
HPG: Hypothalamic-pituitary-gonadal
FSHβ: follicle-stimulating hormone β
LHβ: Luteinizing hormone β
GnRHR: gonadotropin-releasing hormone receptor
FSHR: Follicle-stimulating hormone receptor
LHR: Luteinizing hormone receptor
StAR: steroidogenic acute regulatory protein
3βHSD: 3β-hydroxysteroid dehydrogenase
11βHSD: 11β-hydroxysteroid dehydrogenase
20βHSD: 20β-hydroxysteroid dehydrogenase
17βHSD: 17β-hydroxysteroid dehydrogenase
CYP450: cytochrome P450
GTHα: Gonadotropin Alpha Subunit
LHβ: Luteinizing Hormone Beta Subunit.
PO: Perinuclear oocytes (POs),
COs: Cortical vesicle oocytes
EVs: Early vitellogenic oocytes
LVs: Late vitellogenic oocytes
VTG: Vitellogenin
CHG: choriogenin (CHG)
IL: Interleukin
PC-NPs: Polycarbonate nanoplastics
TRAP: Tartrate-resistant acid phosphatase
LOEC: Lowest observed effect concentration
PAHs: Polycyclic aromatic hydrocarbons
PCBs: Polychlorinated biphenyls
PBDEs: Polybrominated diphenyl ethers
PTFE: Polytetrafluoroethylene (PTFE)

## References

[B1] Abad Lopez A. P. Trilleras J. Arana V. A. Garcia-Alzate L. S. Grande-Tovar C. D. (2023). Atmospheric microplastics: exposure, toxicity, and detrimental health effects. RSC Adv. 13 (11), 7468–7489. 10.1039/d2ra07098g 36908531 PMC9993231

[B2] Abidin U. F. U. Z. Sanny M. Abedin N. H. Z. (2022). Knowledge, attitude, and practice (KAP) of polystyrene food packaging usage among food operators. Food Saf. Pract. Restaurant Industry, IGI Glob., 100–122. 10.4018/978-1-7998-7415-7.ch005

[B3] Abihssira-García I. S. Park Y. Kiron V. Olsvik P. A. (2020). Fluorescent microplastic uptake by immune cells of Atlantic salmon (Salmo salar L.). Front. Environ. Sci. 8, 560206. 10.3389/fenvs.2020.560206

[B4] Akhtar A. Saqib M. A. Tayyaba M. Sohail M. Riaz M. N. Chaudhry A. (2024). Studies on the effect of microplastics (polyethylene glycol) on the growth performance and haematology of Labeo rohita. J. Popul. Ther. Clin. Pharmacol. 31 (4), 1004–1010. 10.53555/jptcp.v31i4.5616

[B5] Alomar C. Sureda A. Capo X. Guijarro B. Tejada S. Deudero S. (2017). Microplastic ingestion by Mullus surmuletus Linnaeus, 1758 fish and its potential for causing oxidative stress. Environ. Res. 159, 135–142. 10.1016/j.envres.2017.07.043 28800471

[B6] Arya G. C. Sarkar S. Manasherova E. Aharoni A. Cohen H. (2021). The plant cuticle: an ancient guardian barrier set against long-standing rivals. Front. Plant Sci. 12, 663165. 10.3389/fpls.2021.663165 34249035 PMC8267416

[B7] Assas M. Qiu X. Chen K. Ogawa H. Xu H. Shimasaki Y. (2020). Bioaccumulation and reproductive effects of fluorescent microplastics in medaka fish. Mar. Pollut. Bull. 158, 111446. 10.1016/j.marpolbul.2020.111446 32753222

[B8] Au S. Y. Lee C. M. Weinstein J. E. van den Hurk P. Klaine S. J. (2017). Trophic transfer of microplastics in aquatic ecosystems: identifying critical research needs. Integr. Environ. Assess. Manag. 13 (3), 505–509. 10.1002/ieam.1907 28440939

[B9] Azarm-Karnagh S. Sattari M. Banaee M. Hadavand B. S. Falco F. (2025). Effects of polystyrene nanoplastics on oxidative stress, blood biochemistry, and digestive enzyme activity in Goldfish (Carassius auratus). Toxics 13 (5), 336. 10.3390/toxics13050336 40423415 PMC12115821

[B10] Bastante-Rabadan M. Boltes K. (2024). Mixtures of Micro and nanoplastics and contaminants of emerging concern in environment: what we know about their toxicological effects. Toxics 12 (8), 589. 10.3390/toxics12080589 39195691 PMC11359687

[B11] Beitsayah A. Hedayati A. Banaee M. Khodadoost S. Safari R. Salati A. (2025). Toxicity impact of polyethylene microplastics on biochemical parameters and oxidative stress in Benni Fish (Barbus sharpeyi). Water Air Soil Pollut. 236, 380. 10.1007/s11270-025-08042-7

[B12] Benson N. U. Agboola O. D. Fred-Ahmadu O. H. Dela-Torre G. E. Oluwalana A. Williams A. B. (2022). Micro (nano) plastics prevalence, food web interactions, and toxicity assessment in aquatic organisms: a review. Front. Mar. Sci. 9, 851281. 10.3389/fmars.2022.851281

[B13] Bhagat J. Zang L. Nishimura N. Shimada Y. (2020). Zebrafish: an emerging model to study microplastic and nanoplastic toxicity. Sci. Total Environ. 728, 138707. 10.1016/j.scitotenv.2020.138707 32361115

[B14] Bhagat J. Nishimura N. Shimada Y. (2021). Toxicological interactions of microplastics/nanoplastics and environmental contaminants: current knowledge and future perspectives. J. Hazard Mater. 405, 123913. 10.1016/j.jhazmat.2020.123913 33127190

[B15] Bhattacharya P. Lin S. Turner J. P. Ke P. C. (2010). Physical adsorption of charged plastic nanoparticles affects algal photosynthesis. J. Phys. Chem. C 114 (39), 16556–16561. 10.1021/jp1054759

[B16] Bhattacharyya S. Greer M. Salehi M. (2025). Impact of micro- and nanoplastics exposure on human health: focus on neurological effects from ingestion. Front. Public Health 13, 1681776. 10.3389/fpubh.2025.1681776 41246084 PMC12616133

[B17] Blonc M. Ruiz N. Balasch J. C. Llorca M. Farre M. Tvarijonaviciute A. (2023). Evaluation of a chronic exposure to nanoplastics in goldfish (carassius auratus): analytical validation of automated assays for the measurement. Ecol. Indic. 147 (11), 109966. 10.1016/j.ecolind.2023.109966

[B18] Bon C. Maretti A. Laura Pulze L. Nicolò Paris N. Santoro O. Pragliola S. (2025). The exposure to polypropylene Micro- and nanoplastics impairs wound healing and tissue regeneration in the leech hirudo verbena. Microplastics 4, 56. 10.3390/microplastics4030056

[B19] Boots B. Russell C. W. Green D. S. (2019). Effects of microplastics in soil ecosystems: above and below ground. Environ. Sci. Techno,l 53 (19), 11496–11506. 10.1021/acs.est.9b03304 31509704

[B20] Britt K. L. Saunders P. K. McPherson S. J. Misso M. L. Simpson E. R. Findlay J. K. (2004). Estrogen actions on follicle formation and early follicle development. Biol. Reprod. 71 (5), 1712–1723. 10.1095/biolreprod.104.028175 15269096

[B21] Bunge A. Kammann U. Scharsack J. P. (2021). Exposure to microplastic fibers does not change fish early life stage development of three-spined sticklebacks (Gasterosteus aculeatus). Microplastics Nanoplastics 1, 15. 10.1186/s43591-021-00015-x

[B22] Cao X. Xie W. Feng M. Chen J. Zhang J. Luo J. (2024). Nanoplastic exposure mediates neurodevelopmental toxicity by activating the oxidative stress response in zebrafish (Danio rerio). ACS Omega 9 (14), 16508–16518. 10.1021/acsomega.4c00231 38617687 PMC11007712

[B23] Chaudhary H. D. Shah G. Bhatt U. Singh H. Soni V. (2025). Microplastics and plant health: a comprehensive review of sources, distribution, toxicity and remediation. Npj Emerg. Contam. 1, 8. 10.1038/s44454-025-00007-z

[B24] Chen J.-C. Chen M.-Y. Fang C. Zheng R.-H. Jiang Y.-L. Zhang Y.-S. (2020). Microplastics negatively impact embryogenesis and modulate the immune response of the marine medaka Oryzias melastigma. Mar. Pollut. Bull. 158, 111349. 10.1016/j.marpolbul.2020.111349 32573451

[B25] Chen F. Aqeel M. Khalid N. Irshad M. K. Farhat F. Nazir A. (2023). Glutathione treatment suppresses the adverse effects of microplastics in rice. Chemosphere 322, 138079. 10.1016/j.chemosphere.2023.138079 36775030

[B26] Chen J.-C. Fang C. Zheng R.-H. Chen M.-L. Kim D.-H. Young-Hwan L. (2022). Environmentally relevant concentrations of microplastics modulated the immune response and swimming activity and impaired the development of marine medaka Oryzias melastigma larvae. Ecotoxicol. Environ. Saf. 241, 113843. 10.1016/j.ecoenv.2022.113843 36068765

[B27] Chen S. Feng Y. Han L. Li D. Feng Y. Jeyakumar P. (2022). Responses of rice (Oryza sativa L.) plant growth, grain yield and quality, and soil properties to the microplastic occurrence in paddy soil. Soils, Sec-2 Global Change. Environ. Risk Assess. Sustain. Land Use 22, 2174–2183. 10.1007/s11368-022-03232-w

[B28] Cheng H. Duan Z. Wu Y. Wang Y. Zhang H. Shi Y. (2022). Immunotoxicity responses to polystyrene nanoplastics and their related mechanisms in the liver of zebrafish (Danio rerio) larvae. Environ. Int. 161, 107128. 10.1016/j.envint.2022.107128 35134711

[B29] Cheng X. Y. Xue H. Wang Z. Ma N. Hu C. Zhang Y. (2025). Maternal exposure to polystyrene nanoplastics during gestation and lactation caused fertility decline in female mouse offspring. Ecotoxicol. Environ. Saf. 289, 117632. 10.1016/j.ecoenv.2024.117632 39755092

[B30] Cocci P. Gabrielli S. Pastore G. Minicucci M. Mosconi G. Palermo F. A. (2022). Microplastics accumulation in gastrointestinal tracts of Mullus barbatus and Merluccius merluccius is associated with increased cytokine production and signaling. Chemosphere 307 (Pt 3), 135813. 10.1016/j.chemosphere.2022.135813 35931257

[B31] Cormier B. Le Bihanic F. Cabar M. Crebassa J.-C. Blanc M. Larsson M. (2021). Chronic feeding exposure to Virgin and spiked microplastics disrupts essential biological functions in teleost fish. J. Hazard. Mater. 415, 125626. 10.1016/j.jhazmat.2021.125626 33740727

[B32] Cui J. Zhang Y. Liu L. Zhang Q. Xu S. Guo M.-Y. (2023). Polystyrene microplastics induced inflammation with activating the TLR2 signal by excessive accumulation of ROS in hepatopancreas of carp (Cyprinus carpio). Ecotoxicol. Environ. Saf. 251, 114539. 10.1016/j.ecoenv.2023.114539 36640574

[B33] D'Avignon G. Wang D. Reid H. B. Gregory-Eaves I. Ricciardi A. (2023). Effects of elevated temperature and microplastic exposure on growth and predatory performance of a freshwater fish. Limnol. Oceanogr. 68 (10), 2245–2260. 10.1002/lno.12417

[B34] Das B. C. Ramanan P. A. Gorakh S. S. Pillai D. Jayadradhan R. V. K. (2023). Sub-Chronic exposure of Oreochromis Niloticus to environmentally relevant concentrations of smaller microplastics: accumulation and toxico-physiological responses. J. Hazard Mater 458, 131916. 10.1016/j.jhazmat.2023.131916 37402322

[B35] De Silva Y. S. K. Rajagopalan U. M. Kodono H. Li D. (2022). Effects of microplastics on lentil (Lens culinaris) seed germination and seedling growth. Chemosphere 303, 135162. 10.1016/j.chemosphere.2022.135162 35654234

[B36] de Souza Machado A. A. Lau C. W. Kloas W. Bergmann J. Bachelier J. B. Faltin E. (2019). Microplastics can change soil properties and affect plant performance. Environ. Sci. Technol. 53 (10), 6044–6052. 10.1021/acs.est.9b01339 31021077

[B37] Del Piano F. Lama A. Piccolo G. Addeo N. F. Iaccarino D. Fusco G. (2023). Impact of polystyrene microplastic exposure on gilthead seabream (Sparus aurata Linnaeus, 1758): differential inflammatory and immune response between anterior and posterior intestine. Sci. Total Environ. 879, 163201. 10.1016/j.scitotenv.2023.163201 37011684

[B38] DiBona E. Haley C. Geist S. Seemann F. (2022). Developmental polyethylenemicroplastic fiber exposure entails subtle reproductive impacts in juvenile Japanese medaka (Oryzias latipes). Environ. Toxicol. Chem. 41, 2848–2858. 10.1002/etc.5456 35942914

[B39] Dong Y. Gao M. Song Z. Qiu W. (2020). Microplastic particles increase arsenic toxicity to rice seedlings. Environ. Pollut. 259, 113892. 10.1016/j.envpol.2019.113892 31931412

[B40] Dong Y. Bao Q. Gao M. Qiu W. Song Z. (2021). A novel mechanism study of microplastic and as co-contamination on indica rice (Oryza sativa L.). J. Hazard. Mater. 421, 126694. 10.1016/j.jhazmat.2021.126694 34332483

[B41] Du J. Xu S. Zhou Q. Li H. Fu L. Tang J. (2020). A review of microplastics in the aquatic environment: distribution, transport, ecotoxicology, and toxicological mechanisms. Environ. Sci. Pollut. Res. 27, 11494–11505. 10.1007/s11356-020-08104-9 32088821

[B42] Elizalde-Velázquez A. Crago J. Zhao X. Green M. J. Cañas-Carrell J. E. (2020). *In vivo* effects on the immune function of fathead minnow (Pimephales promelas) following ingestion and intraperitoneal injection of polystyrene nanoplastics. Sci. Total Environ. 735, 139461. 10.1016/j.scitotenv.2020.139461 32470671

[B43] Espinosa C. Cuesta A. Esteban M. A. (2018). *In vitro* effects of virgin microplastics on fish head-kidney leucocyte activities. Environ. Pollut. 235, 30–38. 10.1016/j.envpol.2017.12.054 29274535

[B44] Espinosa C. Esteban M. A. Cuesta A. (2019). Dietary administration of PVC and PE microplastics: effects on general health, immune status and gene expression in gilthead seabream (Sparus aurata). Fish. Shellfish Immunol. 68, 51–259. 10.1016/j.fsi.2019.10.072 28684324

[B45] Essel R. Engel L. Carus M. Ahrens R. (2015). Sources of microplastics relevant to marine protection in Germany. Texte 64, 31969.

[B46] Evariste L. Barret M. Mottier A. Mouchet F. Gauthier L. Pinelli E. (2019). Gut microbiota of aquatic organisms: a key endpoint for ecotoxicological studies. Environ. Pollut. 248, 989–999. 10.1016/j.envpol.2019.02.101 31091643

[B47] Fang X. Z. Fang S. Q. Ding Y. Ma J. W. Ye Z. Q. Liu D. (2024). Microplastic exposure inhibits nitrate uptake and assimilation in wheat plants. Environ. Pollut. 360, 124626. 10.1016/j.envpol.2024.124626 39084589

[B48] Fernandez-Míguez M. Puvanendran V. Burgerhout E. Presa P. Tveiten H. Vorkamp K. (2023). Effects of weathered polyethylene microplastic ingestion on sexual maturation, fecundity and egg quality in maturing broodstock Atlantic cod (Gadus morhua). Environ. Pollut. 320, 121053. 10.1016/j.envpol.2023.121053 36632969

[B49] Foley C. J. Feiner Z. S. Malinich T. D. Hook T. O. (2018). A meta-analysis of the effects of exposure to microplastics on fish and aquatic invertebrates. Sci. Total Environ. 631–632, 550–559. 10.1016/j.scitotenv.2018.03.046 29529442

[B50] Frank Y. A. Interesova E. A. Solovyev M. M. Xu J. Vorobiev D. S. (2023). Effect of microplastics on the activity of digestive and oxidative-stress-related enzymes in peled whitefish (Coregonus peled Gmelin) larvae. Int. J. Mol. Sci. 24, 10998. 10.3390/ijms241310998 37446176 PMC10341875

[B51] Fu L. Li J. Wang G. Luan Y. Dai W. (2021). Adsorption behavior of organic pollutants on microplastics. Ecotoxicol. Environ. Saf. 217, 112207. 10.1016/j.ecoenv.2021.112207 33866287

[B52] Galloway T. S. (2015). “Micro- and nano-plastics and human health,” in Marine anthropogenic litter (Cham: Springer), 343–366.

[B53] Ghosh T. (2018). An overview on the relevancy between proximate protein content of few fishes with their market price value. J. Aquac. Trop. 32, 385–395.

[B54] Ghosh T. (2025). Microplastics bioaccumulation in fish: its potential toxic effects on hematology, immune response, neurotoxicity, oxidative stress, growth, and reproductive dysfunction. Toxicol. Rep. 14, 101854. 10.1016/j.toxrep.2024.101854 39802604 PMC11720882

[B55] Golwala H. Zhang X. Iskander S. M. Smith A. L. (2021). Solid waste: an overlooked source of microplastics to the environment. Sci. Total Environ. 769, 144581. 10.1016/j.scitotenv.2020.144581 33482549

[B56] Gomes T. Bour A. Coutris C. Almeida A. C. Bråte I. L. N. Wolf R. (2022). “Ecotoxicological impacts of micro- and nanoplastics in terrestrial and aquatic environments,” in Microplastics in the environment: pattern and process. Editor Bank M. S. (Cham: Springer), 199–260.

[B57] González-Doncel M. García-Maurino J. E. Beltrán E. M. Fernández Torija C. Andreu-Sánchez O. Pablos M. V. (2022). Effects of life cycle exposure to polystyrene microplastics on medaka fish (Oryzias latipes). Environ. Pollut. 311, 120001. 10.1016/j.envpol.2022.120001 35995287

[B58] González-Fernández C. Cuesta A. (2022). Nanoplastics increase fish susceptibility to nodavirus infection and reduce antiviral immune responses. Int. J. Mol. Sci. 23, 1483. 10.3390/ijms23031483 35163406 PMC8836078

[B59] Greven A.-C. Merk T. Karagöz F. Mohr K. Klapper M. Jovanović B. (2016). Polycarbonate and polystyrene nanoplastic particles act as stressors to the innate immune system of fathead minnow (Pimephales promelas). Environ. Toxicol. Chem. 35, 3093–3100. 10.1002/etc.3501 27207313

[B60] Gu W. Liu S. Chen L. Liu Y. Gu C. Ren H.-Q. (2020). Single-cell RNA sequencing reveals size-dependent effects of polystyrene microplastics on immune and secretory cell populations from zebrafish intestines. Environ. Sci. Technol. 54, 3417–3427. 10.1021/acs.est.9b06386 32092251

[B61] Guo S. Mu L. Sun S. Hou X. Yao M. Hu X. (2023). Concurrence of microplastics and heat waves reduces rice yields and disturbs the agroecosystem nitrogen cycle. J. Hazard. Mater. 452, 131340. 10.1016/j.jhazmat.2023.131340 37027913

[B62] Gupta R. K. Singh J. M. Leslie T. C. Meachum S. Flaws J. A. Yao H.-C. (2010). Di-(2-ethylhexyl) phthalate and mono-(2-ethylhexyl) phthalate inhibit growth and reduce estradiol levels of antral follicles *in vitro* . Toxicol. Appl. Pharmacol. 242, 224–230. 10.1016/j.taap.2009.10.011 19874833 PMC2789888

[B63] Gupta P. Mahapatra A. Suman A. Ray S. S. Malafaia G. Singh R. K. (2023). Polystyrene microplastics disrupt female reproductive health and fertility *via* SIRT1 modulation in zebrafish (Danio rerio). J. Hazard. Mater. 460, 132359. 10.1016/j.jhazmat.2023.132359 37639793

[B64] Habumugisha T. Zhang Z. Ndayishimiye J. C. Nkinahamira F. Kayiranga A. Cyubahiro E. (2022). Evaluation and optimization of the influence of silver cluster ions on the MALDI-TOF-MS analysis of polystyrene nanoplastic polymers. Anal. Methods 14, 3968–3977. 10.1039/d1ay02219a 35112122

[B65] Habumugisha T. Zhang Z. Uwizewe C. Yan C. Ndayishimiye J. C. Rehman A. (2024). Toxicological review of micro- and nano-plastics in aquatic environments: risks to ecosystems, food web dynamics and human health. Ecotoxicol. Environ. Saf. 278, 116426. 10.1016/j.ecoenv.2024.116426 38718727

[B66] Habumugisha T. Zhang Z. Minor E. C. Rehman A. Yan C. Ndayisenga F. (2026). Micro/nanoplastics as environmental mediators: a systematic review of sources and interfacial processes driving cross-media transport and impacts. J. Hazard. Mater. 504, 141257. 10.1016/j.jhazmat.2026.141257 41619591

[B67] Hamed M. Soliman H. A. M. Osman A. G. M. Sayed A. E.-D. H. (2019). Assessment the effect of exposure to microplastics in Nile tilapia (Oreochromis niloticus) early juvenile: I. Blood biomarkers. Chemosphere 228, 345–350. 10.1016/j.chemosphere.2019.04.153 31039541

[B68] Hamed M. Soliman H. A. M. Osman A. G. M. Sayed A. E.-D. H. (2020). Antioxidants and molecular damage in Nile tilapia (Oreochromis niloticus) after exposure to microplastics. Environ. Sci. Pollut. Res. 27, 14581–14588. 10.1007/s11356-020-07898-y 32048193 PMC7190598

[B69] Hanachi P. Karbalaei S. Walker T. R. Cole M. Hosseini S. V. (2019). Abundance and properties of microplastics found in commercial fish meal and cultured common carp (Cyprinus carpio). Environ. Sci. Pollut. Res. 26, 23777–23787. 10.1007/s11356-019-05637-6 31209753

[B70] Hao Y. Sun Y. Li M. Fang X. Wang Z. Zuo J. (2023). Adverse effects of polystyrene microplastics in the freshwater commercial fish, grass carp (Ctenopharyngodon idella): Emphasis on physiological response and intestinal microbiome. Sci. Total Environ. 856, 159270. 10.1016/j.scitotenv.2022.159270 36208741

[B71] Hara A. Hiramatsu N. Fujita T. (2016). Vitellogenesis and choriogenesis in fishes. Fish. Sci. 82, 187–202. 10.1007/s12562-015-0957-5

[B72] Havixbeck J. J. Barreda D. R. (2015). Neutrophil development, migration, and function in teleost fish. Biology 4, 715–734. 10.3390/biology4040715 26561837 PMC4690015

[B73] He J. Yang X. Liu H. (2021). Enhanced toxicity of triphenyl phosphate to zebrafish in the presence of micro- and nanoplastics. Sci. Total Environ. 756, 143986. 10.1016/j.scitotenv.2020.143986 33307501

[B74] Hong C. Wang Z. Tian M. Wang Y. Liu J. Qiang X. (2024). Migration of microplastics in the rice–duckweed system under different irrigation modes. Agriculture 14, 1460. 10.3390/agriculture14091460

[B75] Horton A. A. Walton A. Spurgeon D. J. Lahive E. Svendsen C. (2017). Microplastics in freshwater and terrestrial environments: evaluating the current understanding to identify the knowledge gaps and future research priorities. Sci. Total Environ. 586, 127–141. 10.1016/j.scitotenv.2017.01.190 28169032

[B76] Hoseinifar S. H. Yousefi S. Doan H. V. Ashouri G. Gioacchini G. Maradonna F. (2021). Oxidative stress and antioxidant defense in fish: the implications of probiotic, prebiotic and synbiotics. Rev. Fish. Sci. Aquac. 29, 198–217. 10.1080/23308249.2020.1795616

[B77] Hu K. Yang Y. Zuo J. Tian W. Wang Y. Duan X. (2022). Emerging microplastics in the environment: properties, distributions, and impacts. Chemosphere 297, 134118. 10.1016/j.chemosphere.2022.134118 35227746

[B78] Hu X. Meng L.-J. Liu H.-D. Guo Y.-S. Liu W.-C. Tan H.-X. (2023). Impacts of Nile tilapia (Oreochromis niloticus) exposed to microplastics in bioflocs system. Sci. Total Environ. 901, 165921. 10.1016/j.scitotenv.2023.165921 37527718

[B79] Huang J.-N. Wen B. Zhu J.-G. Zhang Y.-S. Gao J.-Z. Chen Z.-Z. (2020). Exposure to microplastics impairs digestive performance, stimulates immune response and induces microbiota dysbiosis in the gut of juvenile guppy (Poecilia reticulata). Sci. Total Environ. 733, 138929. 10.1016/j.scitotenv.2020.138929 32466972

[B80] Huang T. Zhao Y. He J. Cheng H. Martyniuk C. J. (2022). Endocrine disruption by azole fungicides in fish: a review of the evidence. Sci. Total Environ. 822, 153412. 10.1016/j.scitotenv.2022.153412 35090921

[B81] Hurley R. R. Nizzetto L. (2018). Fate and occurrence of micro(nano)plastics in soils: knowledge gaps and possible risks. Curr. Opin. Environ. Sci. Health 1, 6–11. 10.1016/j.coesh.2017.10.006

[B82] Hutton S. J. Kashiwabara L. Anderson E. Siddiqui S. Harper B. Harper S. (2024). Behavioral and molecular effects of micro- and nanoplastics across three plastic types in fish: weathered microfibers induce a similar response to nanosized particles. Front. Toxicol. 6, 1490223. 10.3389/ftox.2024.1490223 39659702 PMC11628497

[B83] Iheanacho S. C. Odo G. E. (2020). Neurotoxicity, oxidative stress biomarkers and hematological responses in African catfish (Clarias gariepinus) exposed to polyvinylchloride microparticles. Comp. Biochem. Physiol. C Toxicol. Pharmacol. 232, 108741. 10.1016/j.cbpc.2020.108741 32171890

[B84] Iribarne M. (2021). Inflammation induces zebrafish regeneration. Neural Regen. Res. 16 (9), 1693–1701. 10.4103/1673-5374.306059 33510057 PMC8328752

[B85] Iswahyudi I. Widodo W. Warkoyo W. Sutanto A. Garfansa M. Mujiyanti W. (2024). Investigating the impact of polyethylene, polypropylene, and polystyrene microplastics on seed germination and early growth of rice plants. Environ. Qual. Manag. 34 (1). e22287. 10.1002/tqem.22287

[B86] Jakubowska M. Białowąs M. Stankevičiūtė M. Chomiczewska A. Pažusienė J. Jonko-Sobuś K. (2020). Effects of chronic exposure to microplastics of different polymer types on early life stages of sea trout (Salmo trutta). Sci. Total Environ. 740, 139922. 10.1016/j.scitotenv.2020.139922 32927534

[B87] Jeyavani J. Sibiya A. Stalin T. Vigneshkumar G. Al-Ghanim K. A. Riaz M. N. (2023). Biochemical, genotoxic and histological implications of polypropylene microplastics on freshwater fish Oreochromis mossambicus. Toxics 11, 282. 10.3390/toxics11030282 36977047 PMC10052786

[B88] Jia L. Liu L. Zhang Y. Fu W. Liu X. Wang Q. (2023). Microplastic stress in plants: effects on plant growth and their remediations. Front. Plant Sci. 14, 1226484. 10.3389/fpls.2023.1226484 37636098 PMC10452891

[B89] Jiang X. Chen H. Liao Y. Ye Z. Li M. Klobučar G. (2019). Ecotoxicity and genotoxicity of polystyrene microplastics on higher plant Vicia faba. Environ. Pollut. 250, 831–838. 10.1016/j.envpol.2019.04.055 31051394

[B90] Jiang H. Su J. Zhang Y. Bian K. Wang Z. Wang H. (2022). Insight into the microplastics release from disposable face mask: simulated environment and removal strategy. Chemosphere 309, 136748. 10.1016/j.chemosphere.2022.136748 36209868 PMC9535493

[B91] Jiao S. Shen Z. Fang Q. Liu X. Hao Y. Kong Y. (2025). Toxic effects of microplastics on freshwater fish (Channa argus): mechanisms of inflammation, apoptosis, and autophagy. Aquat. Toxicol. 286, 107450. 10.1016/j.aquatox.2025.107450 40570606

[B92] Jo A.-H. Yu Y.-B. Choi J.-H. Lee J.-H. Choi C.-Y. Kang J.-C. (2025). Microplastics induce toxic effects in fish: bioaccumulation, hematological parameters and antioxidant responses. Chemosphere 375, 144253. 10.1016/j.chemosphere.2025.144253 40022860

[B93] Kabat A. M. Srinivasan N. Maloy K. J. (2014). Modulation of immune development and function by intestinal microbiota. Trends Immunol. 35, 507–517. 10.1016/j.it.2014.07.010 25172617 PMC6485503

[B94] Kadac-Czapska K. Bochentyn B. Maślarz A. Mahlik S. Grembecka M. (2024). Methodology approach for microplastics isolation from samples containing sucrose. Molecules 29, 3996. 10.3390/molecules29173996 39274843 PMC11396657

[B95] Kajal S. Thakur S. (2024). Coexistence of microplastics and heavy metals in soil: occurrence, transport, key interactions and effect on plants. Environ. Res. 262 (Pt 2), 119960. 10.1016/j.envres.2024.119960 39251180

[B96] Kamalakannan M. Rajendran D. Thomas J. Chandrasekaran N. (2024). Synergistic impact of nanoplastics and nanopesticides on Artemia salina and toxicity analysis. Nanoscale Adv. 6, 3119–3134. 10.1039/d4na00013g 38868821 PMC11166108

[B97] Karami A. Romano N. Galloway T. Hamzah H. (2016). Virgin microplastics cause toxicity and modulate the impacts of phenanthrene on biomarker responses in African catfish (Clarias gariepinus). Environ. Res. 151, 58–70. 10.1016/j.envres.2016.07.024 27451000

[B98] Katsumi N. Kusube T. Nagao S. Okochi H. (2021). The input–output balance of microplastics derived from coated fertilizer in paddy fields and the timing of their discharge during the irrigation season. Chemosphere 279, 130574. 10.1016/j.chemosphere.2021.130574 33887593

[B99] Kaur M. Shen C. Wang L. Xu M. (2022). Exploration of single and co-toxic effects of polypropylene microplastics and cadmium on rice (Oryza sativa L.). Nanomaterials 12 (22), 3967. 10.3390/nano12223967 36432253 PMC9696531

[B100] Kelly E. R. M. Trujillo J. E. Setiawan A. Pether S. Burritt D. Allan B. J. M. (2024). Investigating the metabolic and oxidative stress induced by biofouled microplastics exposure in Seriola lalandi (yellowtail kingfish). Mar. Pollut. Bull. 203, 116438. 10.1016/j.marpolbul.2024.116438 38749154

[B101] Khalid N. Aqeel M. Noman A. (2020). Microplastics could be a threat to plants in terrestrial systems directly or indirectly. Environ. Pollut. 267, 115653. 10.1016/j.envpol.2020.115653 33254725

[B102] Khalid N. Aqeel M. Noman A. Khan S. M. Akhter N. (2021). Interactions and effects of microplastics with heavy metals in aquatic and terrestrial environments. Environ. Pollut. 290, 118104. 10.1016/j.envpol.2021.118104 34500399

[B103] Khanashyam A. C. Shanker M. A. Nirmal N. P. (2023). Nano/micro-plastics: sources, trophic transfer, toxicity to animals and humans, regulation, and assessment. Adv. Food Nutr. Res. 103, 141–174. 10.1016/bs.afnr.2022.07.003 36863834

[B104] Kim J. H. Yu Y. B. Choi J. H. (2021). Toxic effects on bioaccumulation, hematological parameters, oxidative stress, immune responses and neurotoxicity in fish exposed to microplastics: a review. J. Hazard. Mater. 413, 125423. 10.1016/j.jhazmat.2021.125423 33930961

[B105] Kim M. J. Kim J. A. Song J. A. Kho K. H. Choi C. Y. (2023). Synthetic microfiber exposure negatively affects reproductive parameters in male medaka (Oryzias latipes). Gen. Comp. Endocrinol. 334, 114216. 10.1016/j.ygcen.2023.114216 36681254

[B106] Köktürk M. Özgeriş F. B. Atamanalp M. Ucar A. Ozdemir S. Parlak V. (2024). Microplastic-induced oxidative stress response in turbot and potential intake by humans. Drug Chem. Toxicol. 47 (3), 296–305. 10.1080/01480545.2023.2168690 36656072

[B107] Kole P. J. Löhr A. J. Van Belleghem F. G. Ragas A. M. (2017). Wear and tear of tyres: a stealthy source of microplastics in the environment. Int. J. Environ. Res. Public Health 14 (10), 1265. 10.3390/ijerph14101265 29053641 PMC5664766

[B108] Kumar M. Xiong X. He M. Tsang D. C. W. Gupta J. Khan E. (2020). Microplastics as pollutants in agricultural soils. Environ. Pollut. 265 (Pt A), 114980. 10.1016/j.envpol.2020.114980 32544663

[B109] Kumar V. Singh E. Singh S. Pandey A. Bhargava P. C. (2023). Micro- and nano-plastics as emerging pollutants in groundwater: environmental impact, potential risks, limitations and way forward towards sustainable management. Chem. Eng. J. 459, 141568. 10.1016/j.cej.2023.141568

[B110] Kumar N. Thorat S. T. Gunaware M. A. Kumar P. Reddy K. S. (2024). Unraveling gene regulation mechanisms in fish: insights into multistress responses and mitigation through iron nanoparticles. Front. Immunol. 15, 1410150. 10.3389/fimmu.2024.1410150 38947331 PMC11211354

[B111] La Pa F. Fasciolo G. Lucariello D. Motta C. M. Venditti P. Ferrandino I. (2024). Polystyrene microplastics effects on zebrafish embryological development: comparison of two different sizes. Environ. Toxicol. Pharmacol. 106, 104371. 10.1016/j.etap.2024.104371 38244881

[B112] Lai W. Xu D. Li J. Wang Z. Ding Y. Wang X. (2021). Dietary polystyrene nanoplastics exposure alters liver lipid metabolism and muscle nutritional quality in large yellow croaker (Larimichthys crocea). J. Hazard. Mater. 419, 126454. 10.1016/j.jhazmat.2021.126454 34198221

[B113] Laily A. N. Fadjar M. Kilawati Y. (2023). Effect of microplastic exposure on male gonad histology of catfish (Clarias gariepinus). J. Aquac. Fish. Health 12 (1), 94–104. 10.20473/jafh.v12i1.36877

[B114] Lee S.-A. Esterhuizen M. Kim Y. Kim M. Kim Y.-J. (2025). Assessing the acute differential toxicity of polystyrene microplastic particles and comparing bead-shaped *versus* fragmented particles on Daphnia magna. Appl. Biol. Chem. 68 (1), 34. 10.1186/s13765-025-01012-x

[B225] Li C. H. Sun H. R. Shi Y. L. Zhao Z. X. Zhang Z. Zhao P. (2023). Polyethylene and poly (butyleneadipate-coterephthalate)-based biodegradable microplastics modulate the bioavailability and speciation of Cd and as in soil: insights into transformation mechanisms. J. Hazard. Mater. 445, 130638. 10.1016/j.jhazmat.2022.130638 37056010

[B115] Li L. Zhou Q. Yin N. Tu C. Luo Y. (2019). Uptake and accumulation of microplastics in an edible plant. Chin. Sci. Bull. 64 (9), 928–934. 10.1360/n972018-00845

[B116] Li L. Luo Y. Li R. Zhou Q. Peijnenburg W. J. Yin N. (2020). Effective uptake of submicrometre plastics by crop plants *via* a crack-entry mode. Nat. Sustain. 3, 929–937. 10.1038/s41893-020-0567-9

[B117] Li H. Liu H. Bi L. Liu Y. Jin L. Peng R. (2024). Immunotoxicity of microplastics in fish. Fish. Shellfish Immunol. 150, 109619. 10.1016/j.fsi.2024.109619 38735599

[B118] Li Y. Li P. Wang W. Zhang Y. Li S. Li D. (2025). Nanoparticle-based drug delivery systems targeting inflammatory immune mechanisms in acute myocardial infarction: current advances and perspectives. Front. Cardiovasc Med. 12, 1657300. 10.3389/fcvm.2025.1657300 41210329 PMC12589070

[B119] Liao H. Gao D. Junaid M. Liu S. Kong C. Chen X. (2023). Parental exposure to polystyrene nanoplastics and di-(2-ethylhexyl) phthalate induces transgenerational growth and reproductive impairments in Daphnia magna. Sci. Total Environ. 882, 1636. 10.1016/j.scitotenv.2023.163657 37084918

[B120] Lin X. Wang Y. Yang X. Watson P. Yang F. Liu H. (2023). Endocrine-disrupting and reproductive toxicity of nano-polystyrene and diethylstilbestrol co-exposure in zebrafish. Sci. Total Environ. 865, 161100. 10.1016/j.scitotenv.2022.161100 36566849

[B121] Liu Y. Guo R. Zhang S. Sun Y. Wang F. (2022a). Uptake and translocation of nano/microplastics by rice seedlings: evidence from a hydroponic experiment. J. Hazard. Mater. 421, 126700. 10.1016/j.jhazmat.2021.126700 34332487

[B122] Liu Y. Zhang J. Zhao H. Cai J. Sultan Y. Fang H. (2022b). Effects of polyvinyl chloride microplastics on reproduction, oxidative stress and detoxification-related genes in Daphnia magna. Comp. Biochem. Physiol. C Toxicol. Pharmacol. 254, 109269. 10.1016/j.cbpc.2022.109269 35026397

[B123] Liu X. Liang C. Fan J. Zhou M. Chang Z. Li L. (2023). Polyvinyl chloride microplastics induce changes in gene expression and organ histology along the HPG axis in Cyprinus carpio larvae. Aquat. Toxicol. 258, 106483. 10.1016/j.aquatox.2023.106483 37023657

[B124] Lu H. Liu L. (2021). Effect of microplastics on the growth of Paralichthys olivaceus. E3S Web Conf. 251, 02040. 10.1051/e3sconf/202125102040

[B125] Lusher A. L. McHugh M. Thompson R. C. (2013). Occurrence of microplastics in the gastrointestinal tract of pelagic and demersal fish from the English Channel. Mar. Pollut. Bull. 67 (1–2), 94–99. 10.1016/j.marpolbul.2012.11.028 23273934

[B126] Lusher A. L. Hollman P. C. H. Mendoza-Hill J. J. (2017a). Microplastics in fisheries and aquaculture: status of knowledge on their occurrence and implications for aquatic organisms and food safety. FAO Fish. Aquac. Tech. Pap.

[B127] Lusher A. L. Welden N. A. Sobral P. Cole M. (2017b). Sampling, isolating and identifying microplastics ingested by fish and invertebrates. Anal. Methods 9 (9), 1346–1360. 10.1039/c6ay02415g

[B128] Lv J. Christie P. Zhang S. (2019). Uptake, translocation, and transformation of metal-based nanoparticles in plants: recent advances and methodological challenges. Environ. Sci. Nano 6, 41–59. 10.1039/c8en00645h

[B129] Ma C. Chen Q. Li J. Li B. Liang W. Su L. (2021). Distribution and translocation of micro- and nanoplastics in fish. Crit. Rev. Toxicol. 51 (9), 740–753. 10.1080/10408444.2021.2024495 35166176

[B130] Ma Y. Li C. Xu X. Zhang Z. Shi H. (2021). Influence of microplastics on the accumulation and chronic toxic effects of cadmium in zebrafish (*Danio rerio*). Chemosphere 202, 514–520. 10.1016/j.chemosphere.2018.03.145 29587232

[B131] Mahmood M. Hussain S. M. Sarker P. K. Ali S. Arif M. S. Nazish N. (2024). Toxicological assessment of dietary exposure of polyethylene microplastics on growth, nutrient digestibility, carcass and gut histology of Nile tilapia (Oreochromis niloticus) fingerlings. Ecotoxicology 33 (3), 296–304. 10.1007/s10646-024-02749-9 38498245

[B132] Malafaia G. Nóbrega R. H. da Luz T. M. da Costa Araújo A. P. (2022). Shedding light on the impacts of gestational exposure to polystyrene nanoplastics on the reproductive performance of Poecilia reticulata females and biochemical responses of embryos. J. Hazard. Mater. 427, 127873. 10.1016/j.jhazmat.2021.127873 34863562

[B133] Mallik A. Xavier K. A. M. Naidu B. C. Nayak B. B. (2021). Ecotoxicological and physiological risks of microplastics on fish and their possible mitigation measures. Sci. Total Environ. 779, 146433. 10.1016/j.scitotenv.2021.146433 33743469

[B134] Mamathaxim N. Song W. Wang Y. Habibul N. (2023). Effects of microplastics on arsenic uptake and distribution in rice seedlings. Sci. Total Environ. 862, 160837. 10.1016/j.scitotenv.2022.160837 36509273

[B135] Mannering A. M. Burritt D. J. Komyakova V. Ferrari M. C. O. Vamvounis G. Gulizia A. M. (2025). Microplastic consumption elevates fish oxidative stress but does not affect predator-driven mortality. Sci. Total Environ. 983, 179644. 10.1016/j.scitotenv.2025.179644 40409023

[B136] Martin L. Marbach S. Zimba P. Liu Q. Xu W. (2023). Uptake of nanoplastic particles by zebrafish embryos triggers macrophage responses at early developmental stages. Chemosphere 341, 140069. 10.1016/j.chemosphere.2023.140069 37673181

[B137] Matozzo V. Gagné F. Marin M. G. Ricciardi F. Blaise C. (2008). Vitellogenin as a biomarker of exposure to estrogenic compounds in aquatic invertebrates: a review. Environ. Int. 34, 531–545. 10.1016/j.envint.2007.09.008 18029015

[B138] Menéndez-Pedriza A. Jaumot J. (2020). Interaction of environmental pollutants with microplastics: a critical review of sorption factors, bioaccumulation and ecotoxicological effects. Toxics 8 (2), 40. 10.3390/toxics8020040 32498316 PMC7355763

[B139] Mo Q. Yang X. Wang J. Xu H. Li W. Fan Q. (2021). Adsorption mechanism of two pesticides on polyethylene and polypropylene microplastics: density functional theory calculations and particle size effects. Environ. Pollut. 291, 118120. 10.1016/j.envpol.2021.118120 34520951

[B140] Mohammadzadeh S. Zaretabar A. Ahmadifar E. Khajeh M. Moghadam M. S. Milla S. (2024). Polystyrene nanoplastics disrupt hepatic vitellogenin metabolism and impair reproduction in female zebrafish. Ann. Anim. Sci. 24 (3), 843–849. 10.2478/aoas-2024-0022

[B141] Mustafa S. A. Al-Rudainy A. J. Salman N. M. (2024). Effect of environmental pollutants on fish health: an overview. Egypt. J. Aquat. Res. 50 (2), 225–233. 10.1016/j.ejar.2024.02.006

[B142] Nam S.-E. Haque M. N. Lee S. Kim C. H. Kim T. H. Rhee J.-S. (2024). Negligible additive effects of polyethylene terephthalate microplastics on growth and reproduction of Java medaka exposed to 17β-estradiol and bisphenol A. Aquat. Toxicol. 274, 107052. 10.1016/j.aquatox.2024.107052 39163697

[B143] Ohkubo N. Ito M. Hano T. Kono K. Mochida K. (2020). Estimation of uptake and gut retention of microplastics in juvenile marine fish. Mar. Pollut. Bull. 160, 111630. 10.1016/j.marpolbul.2020.111630 32911116

[B144] Parkin J. Cohen B. (2001). An overview of the immune system. Lancet 357 (9270), 1777–1789. 10.1016/S0140-6736(00)04904-7 11403834

[B145] Pei J. Chen S. Li L. Wang K. Pang A. Niu M. (2024). Impact of polystyrene nanoplastics on apoptosis and inflammation in zebrafish larvae. Sci. Total Environ. 948, 174737. 10.1016/j.scitotenv.2024.174737 39004365

[B146] Pironti C. Ricciardi M. Motta O. Miele Y. Proto A. Montano L. (2021). Microplastics in the environment: intake through the food web, human exposure and toxicological effects. Toxics 9, 224. 10.3390/toxics9090224 34564375 PMC8473407

[B147] Pitt J. A. Kozal J. S. Jayasundara N. Massarsky A. Trevisan R. Geitner N. (2018). Uptake, tissue distribution and toxicity of polystyrene nanoparticles in developing zebrafish (Danio rerio). Aquat. Toxicol. 194, 185–194. 10.1016/j.aquatox.2017.11.017 29197232 PMC6959514

[B148] Qi Y. Yang X. Mejia Pelaez A. Huerta Lwanga E. Beriot N. Gertsen H. (2018). Macro- and microplastics in soil–plant systems: effects on wheat growth. Sci. Total Environ. 645, 1048–1056. 10.1016/j.scitotenv.2018.07.229 30248830

[B149] Qiang L. Cheng J. (2021). Exposure to polystyrene microplastics impairs gonads of zebrafish (Danio rerio). Chemosphere 263, 128161. 10.1016/j.chemosphere.2020.128161 33297137

[B150] Qiao R. Deng Y. Zhang S. Wolosker M. B. Zhu Q. Ren H. (2019). Accumulation of different shapes of microplastics induces intestinal injury and gut microbiota dysbiosis in zebrafish. Chemosphere 236, 124334. 10.1016/j.chemosphere.2019.07.065 31310986

[B151] Qiu S.-Q. Huang G.-Y. Li X.-P. Lei D.-Q. Wang C.-S. Ying G.-G. (2023). Endocrine disruptor responses in marine medaka embryos after exposure to aged plastic leachates. Aquat. Toxicol. 261, 106635. 10.1016/j.aquatox.2023.106635 37478585

[B152] Rajendran D. Chandrasekaran N. (2023). Journey of micronanoplastics with blood components. RSC Adv. 13 (45), 31435–31459. 10.1039/d3ra05620a 37901269 PMC10603568

[B153] Rashid E. Hussain S. M. Kucharczyk D. Nowosad J. Al-Ghanim K. A. Sarker P. K. (2024a). Toxicological consequences of polystyrene microplastics on Cirrhinus mrigala. Mar. Freshw. Res. 75, MF24055. 10.1071/MF24055

[B154] Rashid E. Hussain S. M. Sarker P. K. Ali S. Paray B. A. (2024b). Dietary polystyrene microplastics alter growth and gut histopathology in Catla catla. Aquac. Rep. 36, 102100. 10.1016/j.aqrep.2024.102100

[B155] Rashid E. Hussain S. M. Ali S. Kucharczyk D. Nowosad J. Al-Ghanim K. A. (2025a). Polystyrene microplastics exposure in Labeo rohita: physiological and histopathological evaluation. Sci. Rep. 15, 12888. 10.1038/s41598-025-95811-3 40234586 PMC12000475

[B156] Rashid E. Hussain S. M. Ali S. Kucharczyk D. Nowosad J. Al-Ghanim K. A. (2025b). Physiological and health responses of Catla catla fingerlings to polystyrene microplastics. Sci. Rep. 15 (1), 2218. 10.1038/s41598-025-85291-w 39820509 PMC11739412

[B157] Rehman A. Huang F. Zhang Z. Habumugisha T. Yan C. Shaheen U. (2024). Nanoplastic contamination alters zebrafish liver metabolism. Environ. Int. 187, 108713. 10.1016/j.envint.2024.108713 38703446

[B158] Rehman A. Habumugisha T. Huang F. Zhang Z. Shaheen U. Yan C. (2025). Impacts of polystyrene nanoplastics on zebrafish gut microbiota. Ecotoxicol. Environ. Saf. 299, 118332. 10.1016/j.ecoenv.2025.118332 40393324

[B159] Rillig M. C. Lehmann A. de Souza Machado A. A. Yang G. (2019). Microplastic effects on plants. New Phytol. 223, 1066–1070. 10.1111/nph.15794 30883812

[B160] Rios-Fuster B. Arechavala-Lopez P. García-Marcos K. Alomar C. Compa M. Alvarez E. (2021). Experimental evidence of physiological and behavioral effects of microplastic ingestion in Sparus aurata. Aquat. Toxicol. 231, 105737. 10.1016/j.aquatox.2020.105737 33422861

[B161] Roch S. Rebl A. Wolski W. Brinker A. (2022). Combined proteomic and gene expression analysis to investigate reduced performance in rainbow trout (Oncorhynchus mykiss) caused by environmentally relevant microplastic exposure. Microplast. Nanoplast. 2, 14. 10.1186/s43591-022-00034-2

[B162] Rochman C. M. Hoh E. Kurobe T. Teh S. J. (2013). Ingested plastic transfers hazardous chemicals to fish and induces hepatic stress. Sci. Rep. 3, 3263. 10.1038/srep03263 24263561 PMC3836290

[B163] Roy T. Dey T. K. Jamal M. (2023). Microplastic/nanoplastic toxicity in plants: an imminent concern. Environ. Monit. Assess. 195, 27. 10.1007/s10661-022-10654-z 36279030 PMC9589797

[B164] Ryan P. G. (2019). “Ingestion of plastics by marine organisms,” in Hazardous chemicals associated with plastics in the Marine environment. Editors Takada H. Karapanagioti H. K. (Springer International Publishing), 235–266.

[B165] Sahasa R. G. K. Dhevagi P. Poornima R. Ramya A. Moorthy P. S. Alagirisamy B. (2023). Effect of polyethylene microplastics on seed germination of blackgram (Vigna mungo L.) and tomato (Solanum lycopersicum L.). Environ. Adv. 11, 100349. 10.1016/j.envadv.2023.100349

[B166] Santos D. Félix L. Luzio A. Parra S. Cabecinha E. Bellas J. (2020). Toxicological effects induced on early life stages of zebrafish (Danio rerio) after an acute exposure to microplastics alone or co-exposed with copper. Chemosphere 261, 127748. 10.1016/j.chemosphere.2020.127748 32738713

[B167] Santos D. Luzio A. Félix L. Bellas J. Monteiro S. M. (2022). Oxidative stress, apoptosis and serotonergic system changes in zebrafish (Danio rerio) gills after long-term exposure to microplastics and copper. Comp. Biochem. Physiol. C Toxicol. Pharmacol. 258, 109363. 10.1016/j.cbpc.2022.109363 35525464

[B168] Sarasamma S. Audira G. Siregar P. Malhotra N. Lai Y.-H. Liang S.-T. (2020). Nanoplastics cause neurobehavioral impairments, reproductive and oxidative damages, and biomarker responses in zebrafish. Int. J. Mol. Sci. 21, 1410. 10.3390/ijms21041410 32093039 PMC7073134

[B169] Scanes E. Wood H. Ross P. (2019). Microplastics detected in haemolymph of the Sydney rock oyster (Saccostrea glomerata). Mar. Pollut. Bull. 149, 110537. 10.1016/j.marpolbul.2019.110537 31466014

[B170] Sener I. Zarantoniello M. Cattaneo N. Conti F. Succi L. Chemello G. (2025). Mitigation of dietary microplastic accumulation and oxidative stress response in rainbow trout (Oncorhynchus mykiss) fry through dietary supplementation of a natural microencapsulated antioxidant. Anim. (Basel) 15, 1020. 10.3390/ani15071020 40218413 PMC11988097

[B171] Shen M. Zeng G. Zhang Y. Wen X. Song B. Tang W. (2019). Can biotechnology strategies effectively manage environmental (micro)plastics? Sci. Total Environ. 697, 134200. 10.1016/j.scitotenv.2019.134200 31491631

[B172] Smith G. S. Lumsden J. H. (1983). Review of neutrophil adherence, chemotaxis, phagocytosis and killing. Vet. Immunol. Immunopathol. 4 (1–2), 177–236. 10.1016/0165-2427(83)90058-2 6346663

[B173] Solomando A. Capo X. Alomar C. Alvarez E. Compa M. Valencia J. M. (2020). Long-term exposure to microplastics induces oxidative stress and a pro-inflammatory response in the gut of Sparus aurata Linnaeus, 1758. Environ. Pollut. 266, 115295. 10.1016/j.envpol.2020.115295 32763772

[B174] Solomando A. Capo X. Alomar C. Compa M. Valencia J. M. Sureda A. (2021). Assessment of the effect of long-term exposure to microplastics and depuration period in Sparus aurata Linnaeus, 1758: liver and blood biomarkers. Sci. Total Environ. 786, 147479. 10.1016/j.scitotenv.2021.147479 33975116

[B175] Spano C. Muccifora S. Castiglione M. R. Bellani L. Bottega S. Giorgetti L. (2022). Polystyrene nanoplastics affect seed germination, cell biology and physiology of rice seedlings in short-term treatments: evidence of their internalization and translocation. Plant Physiol. biochem. 172, 158–166. 10.1016/j.plaphy.2022.01.012 35074726

[B176] Stapleton M. J. Hai F. I. (2023). Microplastics as an emerging contaminant of concern to our environment: a brief overview of the sources and implications. Bioengineered 8 (1), 2244754. 10.1080/21655979.2023.2244754 37553794 PMC10413915

[B177] Sun Z. Wu B. Yi J. Yu H. He J. Teng F. (2024). Impacts of environmental concentrations of nanoplastics on zebrafish neurobehavior and reproductive toxicity. Toxics 12 (8), 617. 10.3390/toxics12080617 39195719 PMC11359748

[B178] Sun H H. Shi Y. Zhao P. Long G. Li C. Wang J. (2023). Effects of polyethylene and biodegradable microplastics on photosynthesis, antioxidant defense systems, and arsenic accumulation in maize (Zea mays L.) seedlings grown in arsenic-contaminated soils. Sci. Total Environ. 868, 161557. 10.1016/j.scitotenv.2023.161557 36640877

[B179] Sun N N. Shi H. Li X. Gao C. Liu R. (2023). Combined toxicity of micro/nanoplastics loaded with environmental pollutants to organisms and cells: Role, effects, and mechanism. Environ. Int. 171, 107711. 10.1016/j.envint.2022.107711 36566717

[B180] Sussarellu R. Suquet M. Thomas Y. Lambert C. Fabioux C. Pernet M. E. J. (2016). Oyster reproduction is affected by exposure to polystyrene microplastics. Proc. Natl. Acad. Sci. U.S.A. 113, 2430–2435. 10.1073/pnas.1519019113 26831072 PMC4780615

[B226] Tan J. Chen Y. Mo Z. Tan C. Wen R. Chen Z. (2022). Zinc oxide nanoparticles and polyethylene microplastics affect the growth, physiological and biochemical attributes, and Zn accumulation of rice seedlings. Environ. Sci. Pollut. Res. Int. 29 (40), 61534–61546. 10.1007/s11356-022-19262-3 35445922

[B181] Tanaka K. Takada H. (2016). Microplastic fragments and microbeads in digestive tracts of planktivorous fish from urban coastal waters. Sci. Rep. 6, 34351. 10.1038/srep34351 27686984 PMC5043373

[B182] Tang K. H. D. (2025). Combined toxicity of microplastics and antimicrobials on animals: a review. Antibiot. (Basel) 14 (9), 896. 10.3390/antibiotics14090896 41009875 PMC12466353

[B183] Tang M. Huang Y. Zhang W. Fu T. Zeng T. Huang Y. (2022). Effects of microplastics on the mineral elements absorption and accumulation in hydroponic rice seedlings (Oryza sativa L.). Bull. Environ. Contam. Toxicol. 108, 949–955. 10.1007/s00128-021-03453-8 35079849

[B184] Taylor S. E. Pearce C. I. Sanguinet K. A. Hu D. Chrisler W. B. Kim Y. (2020). Polystyrene nano- and microplastic accumulation at Arabidopsis and wheat root cap cells, but no evidence for uptake into roots. Environ. Sci. Nano 7, 1942–1953. 10.1039/d0en00309c

[B185] Thapliyal C. Negi S. Nagarkoti S. Daverey A. (2025). Mechanistic insight into potential toxic effects of microplastics and nanoplastics on human health. Discov. Appl. Sci. 7, 645. 10.1007/s42452-025-07214-8

[B186] Thomas P. J. Perono G. Tommasi F. Pagano G. Oral R. Burić P. (2021). Resolving the effects of environmental micro- and nanoplastics exposure in biota: a knowledge gap analysis. Sci. Total Environ. 780, 146534. 10.1016/j.scitotenv.2021.146534 34030291

[B187] Thompson R. C. Swan S. H. Moore C. J. Vom Saal F. S. (2009). Our plastic age. Philos. Trans. R. Soc. B Biol. Sci. 364, 1973–1976. 10.1098/rstb.2009.0054 19528049 PMC2874019

[B188] Trivedi A. Bakhasha J. Saxena V. Arya N. Kumar P. Srivastava A. (2026). Microplastic-metal interactions and their toxicological effects in fish: a comprehensive review. J. Hazard. Mater. Adv. 21, 101005. 10.1016/j.hazadv.2026.101005

[B189] Urme S. Shah I. Chowdhury I. (2019). Impact of microplastic in aquaculture: a review.

[B190] Wagner M. Scherer C. Alvarez-Muñoz D. Brennholt N. Bourrain X. Buchinger S. (2014). Microplastics in freshwater ecosystems: what we know and what we need to know. Environ. Sci. Eur. 26, 12. 10.1186/s12302-014-0012-7 28936382 PMC5566174

[B191] Wang J. Li Y. Lu L. Zheng M. Zhang X. Tian H. (2019). Polystyrene microplastics cause tissue damages, sex-specific reproductive disruption and transgenerational effects in marine medaka (Oryzias melastigma). Environ. Pollut. 254, 113024. 10.1016/j.envpol.2019.113024 31454586

[B224] Wang L. Tanveer M. (2023). Editorial to the special issue “Eco-physiological and molecular basis of stress tolerance in plants. Biology 12 (3), 485. 10.3390/biology12030485 36979176 PMC10045121

[B192] Wang Y. Yang Y. Liu X. Zhao J. Liu R. Xing B. (2021). Interaction of microplastics with antibiotics in aquatic environment: distribution, adsorption, and toxicity. Environ. Sci. Technol. 55, 15579–15595. 10.1021/acs.est.1c04509 34747589

[B193] Wang J. Li X. Gao M. Li X. Zhao L. Ru S. (2022). Polystyrene microplastics increase estrogenic effects of 17α-ethynylestradiol on male marine medaka (Oryzias melastigma). Chemosphere 287, 132312. 10.1016/j.chemosphere.2021.132312 34563785

[B194] Wang J. Wu F. Dong S. Wang X. Ai S. Liu Z. (2024). Meta-analysis of the effects of microplastic on fish: insights into growth, survival, reproduction, oxidative stress, and gut Microbiota diversity. Water Res. 267, 122493. 10.1016/j.watres.2024.122493 39321729

[B195] Wang T. Yu C. Chu Q. Wang F. Lan T. Wang J. (2020). Adsorption behavior and mechanism of five pesticides on microplastics from agricultural polyethylene films. Chemosphere 244, 125491. 10.1016/j.chemosphere.2019.125491 31835051

[B196] Wang W. Ge J. Yu X. (2020). Bioavailability and toxicity of microplastics to fish species: a review. Ecotoxicol. Environ. Saf. 189, 109913. 10.1016/j.ecoenv.2019.109913 31735369

[B197] Watts A. J. Lewis C. Goodhead R. M. Beckett S. J. Moger J. Tyler C. R. (2014). Uptake and retention of microplastics by the shore crab Carcinus maenas. Environ. Sci. Technol. 48, 8823–8830. 10.1021/es501090e 24972075

[B198] Wei W. Yang Q. Xiang D. Chen X. Wen Z. Wang X. (2023). Combined impacts of microplastics and cadmium on the liver function, immune response, and intestinal microbiota of crucian carp (Carassius carassius). Ecotoxicol. Environ. Saf. 261, 115104. 10.1016/j.ecoenv.2023.115104 37295303

[B199] Weichselbaum L. Klein O. D. (2018). The intestinal epithelial response to damage. Sci. China Life Sci. 61, 1205–1211. 10.1007/s11427-018-9331-y 30194677

[B200] Worm B. Lotze H. K. Jubinville I. Wilcox C. Jambeck J. (2017). Plastic as a persistent marine pollutant. Annu. Rev. Environ. Resour. 42, 1–26. 10.1146/annurev-environ-102016-060700

[B201] Wu X. Hou H. Liu Y. Yin S. Bian S. Liang S. (2022). Microplastics affect rice (Oryza sativa L.) quality by interfering metabolite accumulation and energy expenditure pathways: a field study. J. Hazard. Mater. 422, 126834. 10.1016/j.jhazmat.2021.126834 34390954

[B202] Wu Y. Zhu J. Sun Y. Wang S. Wang J. Zhang X. (2024). Effects of the co-exposure of microplastic/nanoplastic and heavy metal on plants: using CiteSpace, meta-analysis, and machine learning. Ecotoxicol. Environ. Saf. 286, 117237. 10.1016/j.ecoenv.2024.117237 39447297

[B203] Xia X. Guo W. Ma X. Liang N. Duan X. Zhang P. (2023). Reproductive toxicity and cross-generational effect of polyethylene microplastics in Paramisgurnus dabryanus. Chemosphere 313, 137440. 10.1016/j.chemosphere.2022.137440 36460160

[B204] Xu M. Fang W. Lin G. Zhu X. Lu J. (2023). Physiological and transcriptional effects of polyethylene microplastics exposure on yellowfin seabream (Acanthopagrus latus) larvae in the aquatic environment.

[B205] Yan W. Hamid N. Deng S. Jia P.-P. Pei D.-S. (2020). Individual and combined toxicogenetic effects of microplastics and heavy metals (Cd, Pb, and Zn) perturb gut microbiota homeostasis and gonadal development in marine medaka (Oryzias melastigma). J. Hazard. Mater. 397, 122795. 10.1016/j.jhazmat.2020.122795 32388101

[B206] Yan Z. Zhou Y. Zhu P. Bao X. Su P. (2023). Polystyrene nanoplastics mediated the toxicity of silver nanoparticles in zebrafish embryos. Front. Mar. Sci. 10, 1195125. 10.3389/fmars.2023.1195125

[B207] Yang C. Gao X. (2022). Impact of microplastics from polyethylene and biodegradable mulch films on rice (Oryza sativa L.). Sci. Total Environ. 828, 154579. 10.1016/j.scitotenv.2022.154579 35302020

[B208] Yang Y. Jin Z. Mueller N. D. Driscoll A. W. Hernandez R. R. Grodsky S. M. (2023). Sustainable irrigation and climate feedback. Nat. Food 4, 654–663. 10.1038/s43016-023-00821-x 37591963

[B209] Yaripour H. S. Huuskonen H. Rahman T. Kekäläinen J. Akkanen J. Magris M. (2021). Pre-fertilization exposure of sperm to nano-sized plastic particles decreases offspring size and swimming performance in the European whitefish (Coregonus lavaretus). Environ. Pollut. 291, 118196. 10.1016/j.envpol.2021.118196 34555795

[B210] Yedier S. Yalçınkaya S. K. Bostancı D. (2023). Exposure to polypropylene microplastics *via* diet and water induces oxidative stress in Cyprinus carpio. Aquat. Toxicol. 259, 106540. 10.1016/j.aquatox.2023.106540 37062245

[B211] Yong C. Q. Y. Valiyaveettil S. Tang B. L. (2020). Toxicity of microplastics and nanoplastics in mammalian systems. Int. J. Environ. Res. Public Health 17, 1509. 10.3390/ijerph17051509 32111046 PMC7084551

[B212] Young D. Worrell A. McDevitt E. Henein L. Howell III G. E. (2020). Alterations in macrophage phagocytosis and inflammatory tone following exposure to the organochlorine compounds oxychlordane and trans-nonachlor. Toxicol Vitro 65, 104791. 10.1016/j.tiv.2020.104791 32057836 PMC7152568

[B213] Yu H. Zhang Y. Tan W. Zhange Z. (2022). Microplastics as an emerging environmental pollutant in agricultural soils: effects on ecosystems and human health. Front. Environ. Sci. 10, 855292. 10.3389/fenvs.2022.855292

[B214] Yuan Z. Nag R. Cummins E. (2022). Human health concerns regarding microplastics in the aquatic environment—from marine to food systems. Sci. Total Environ. 823, 153730. 10.1016/j.scitotenv.2022.153730 35143789

[B215] Zhang Q.-F. Li Y.-W. Liu Z.-H. Chen Q.-L. (2016). Reproductive toxicity of inorganic mercury exposure in adult zebrafish: histological damage, oxidative stress, and alterations of sex hormone and gene expression in the hypothalamic–pituitary–gonadal axis. Aquat. Toxicol. 177, 417–424. 10.1016/j.aquatox.2016.06.018 27391360

[B216] Zhang W. Zhang S. Zhao Q. Qu L. Wang J. (2020). Spatio-temporal distribution of plastic and microplastic debris in the surface water of the Bohai Sea, China. Mar. Pollut. Bull. 158, 111343. 10.1016/j.marpolbul.2020.111343 32753167

[B217] Zhang F. Li D. Yang Y. Zhang H. Zhu J. Liu J. (2022). Combined effects of polystyrene microplastics and copper on antioxidant capacity, immune response and intestinal microbiota of Nile tilapia (Oreochromis niloticus). Sci. Total Environ. 808, 152099. 10.1016/j.scitotenv.2021.152099 34863761

[B218] Zhao W. Chen Y. Hu N. Long D. Cao Y. (2024). The uses of zebrafish (Danio rerio) as an *in vivo* model for toxicological studies: a review based on bibliometrics. Ecotoxicol. Environ. Saf. 272, 116023. 10.1016/j.ecoenv.2024.116023 38290311

[B219] Zheng Y. Addotey T. N. A. Chen J. Xu G. (2023). Effect of polystyrene microplastics on the antioxidant system and immune response in GIFT (Oreochromis niloticus). Biol. (Basel) 12, 1430. 10.3390/biology12111430 37998029 PMC10669825

[B220] Zheng Y. Gan X. Lin C. Wang D. Chen R. Dai Y. (2024). Polystyrene nanoplastics cause reproductive toxicity in zebrafish: PPAR-mediated lipid metabolism disorder. Sci. Total Environ. 931, 172795. 10.1016/j.scitotenv.2024.172795 38677429

[B221] Zhenghua Y. Zhenhua Z. Gui C. Zed R. Haijun S. (2023). Microplastics have rice cultivar-dependent impacts on grain yield and quality, and nitrogenous gas losses from paddy, but not on soil properties. J. Hazard. Mater. 446. 10.1016/j.jhazmat.2022.130672 36580778

[B222] Zhou C. Q. Lu C. H. Mai L. Bao L. J. Liu L. Y. Zeng E. Y. (2021). Response of rice (Oryza sativa L.) roots to nanoplastic treatment at seedling stage. J. Hazard. Mater. 401, 123412. 10.1016/j.jhazmat.2020.123412 32763702

[B223] Zhou Y. Jin Q. Xu H. Wang Y. Li M. (2023). Chronic nanoplastic exposure induced oxidative and immune stress in medaka gonad. Sci. Total Environ. 869, 161838. 10.1016/j.scitotenv.2023.161838 36716889

